# From Fasting to Caloric Restriction: State of the Art and Clinical Implications

**DOI:** 10.3390/nu18213660

**Published:** 2026-11-04

**Authors:** Lucio Lucchin, Fabiano Cimmino, Eduardo Penna, Carmela Bagnato, Barbara Paolini, Odette Misa Sonia Hassan, Annalisa Maghetti, Maria Grazia Carbonelli, Ilaria Leone, Marco Salvatore, Marianna Crispino, Giovanna Trinchese, Maria Pina Mollica

**Affiliations:** 1 Dietetics and Clinical Nutrition, Bolzano Health District, 39100 Bolzano, Italy; lucio.lucchin@nucl.it; 2 Department of Biology, University of Naples Federico II, 80126 Naples, Italy; fabiano.cimmino@unina.it (F.C.); eduardo.penna@unina.it (E.P.); giovanna.trinchese@unina.it (G.T.); mariapia.mollica@unina.it (M.P.M.); 3 UOSD Clinical Nutrition and Dietetic, Hospital Matera, 75100 Matera, Italy; drcarmela.bagnato@gmail.com; 4 Dietetics and Clinical Nutrition, Azienda Ospedaliera Universitaria Senese, Policlinico Santa Maria Alle Scotte, 53100 Siena, Italy; info@barbarapaolini.it; 5 S. Camillo-Forlanini Hospital, 00152 Rome, Italy; odette.hassan@gmail.com (O.M.S.H.); mcarbonelli@scf.rm.it (M.G.C.); 6 Centro Specialistico di Dietetica e Nutrizione Clinica Matrik, 40138 Bologna, Italy; a.maghetti@gmail.com; 7 IRCCS Synlab SDN, 80143 Naples, Italy; ilaria.leone@synlab.it (I.L.); direzionescientifica.irccssdn@synlab.it (M.S.)

**Keywords:** fasting, caloric restriction, metabolic health, circadian biology, metabolic flexibility

## Abstract

Fasting and caloric restriction (CR) are central to current nutritional and metabolic research, although the complexity of physiological responses and their clinical applicability have yet to be fully elucidated. This review provides a state-of-the-art overview of fasting-based and CR-based interventions, including prolonged fasting, alternate-day fasting, semi-fasting, fasting-mimicking diets, and time-restricted feeding, with particular emphasis on their role in metabolic health. Mechanistically, these nutritional strategies modulate ketogenesis, autophagy, mitochondrial function, circadian biology, and nutrient-sensing pathways, including AMPK, mTOR, IGF-1, sirtuins, GCN2, and FGF21, thereby shifting cellular programs from growth and substrate storage toward stress resistance, repair, and metabolic flexibility. Current evidence supports beneficial effects of these nutritional interventions on body weight, adiposity, insulin sensitivity, inflammatory tone, and selected cardiometabolic risk factors. Evidence regarding neuroprotection, cancer prevention, and lifespan extension largely comes from preclinical studies, and the responses in humans are still unclear. This review also highlights the relevance of meal timing, refeeding, protein intake, and interindividual variability in shaping physiological responses and clinical outcomes. Overall, fasting and CR should not be viewed as a rigid protocol but as a personalized nutritional framework whose efficacy and safety depend on timing, duration, and patient context.

## 1. Methods

### Literature Search and Selection

This review provides a narrative overview of the current state of research on fasting and caloric restriction (CR), focusing on their physiological mechanisms, metabolic effects, clinical implications, and potential relevance to healthy aging. The literature was identified through searches of the databases PubMed/MEDLINE, Embase, Scopus, and Web of Science, covering publications available from 2000 to 2025. The search strategy combined terms related to fasting and dietary restriction, including “fasting”, “intermittent fasting”, “time-restricted feeding”, “time-restricted eating”, “alternate-day fasting”, “prolonged fasting”, “fasting-mimicking diet”, and “caloric restriction”, with terms addressing the main topics covered in the review, including “ketogenesis”, “autophagy”, “mitochondrial function”, “circadian rhythms”, “insulin sensitivity”, “inflammation”, “stem cells”, “neuroprotection”, “cancer”, and “longevity”. Search terms were appropriately combined to identify relevant studies and reviews. The selected literature included randomised controlled trials, other human intervention studies, observational studies, systematic and narrative reviews, meta-analyses, and preclinical investigations involving animal models and in vitro or mechanistic approaches. Studies were selected according to their relevance to the scope of the review, the scientific question addressed, and their contribution to the understanding of the physiological mechanisms, metabolic outcomes, clinical applications, and potential limitations of fasting and CR. Particular attention was given to human studies addressing clinical and metabolic outcomes, while animal and in vitro studies were considered primarily to discuss underlying mechanisms and hypotheses requiring further clinical validation.

## 2. Introduction

**Figure 1:**
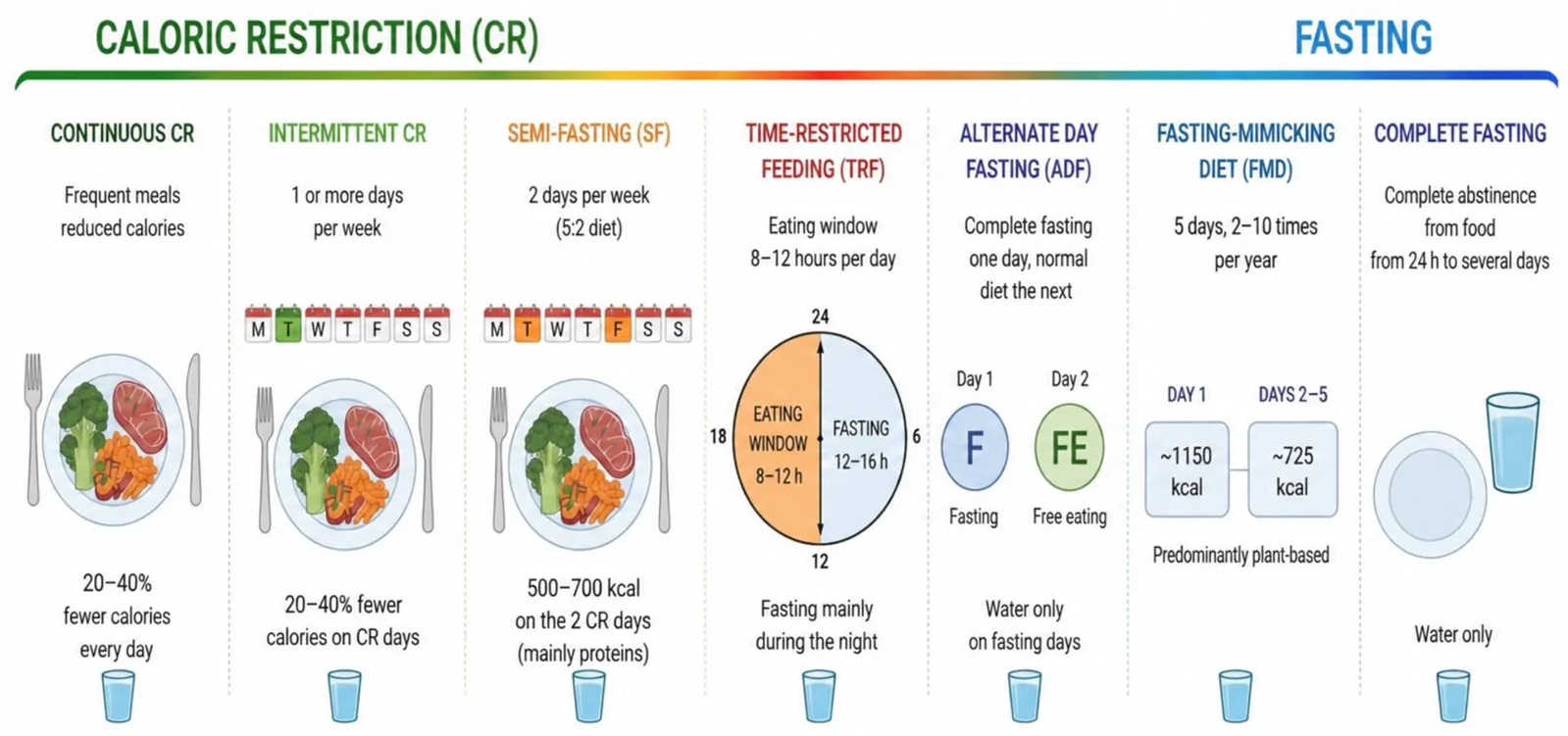
**Dietary Strategies of Caloric Restriction and Fasting: Main Approaches and Feeding Patterns.** Schematic overview of the principal dietary approaches based on caloric restriction (CR) and fasting. Continuous and intermittent CR involve a chronic or periodic reduction in caloric intake, while semi-fasting (SF) typically consists of 500–700 kcal intake on restricted days. Time-restricted feeding (TRF) alternates daily eating and fasting windows, generally within an 8–12 h eating period. Alternate-day fasting (ADF) alternates fasting and unrestricted-feeding days. The fasting-mimicking diet (FMD) is a periodic low-calorie, predominantly plant-based protocol designed to reproduce some metabolic effects of fasting while maintaining limited nutrient intake. Complete fasting involves total abstinence from food, with water allowed.

This review examines the current state of knowledge on fasting and caloric restriction (CR), which are achieving growing popularity and dissemination with their various modalities. The fasting, defined as the complete abstention from food, with only water permitted [1], can last for several consecutive days, or it can be alternated with unrestricted eating, as in Alternate Day Fasting (ADF). On the other hand, CR involves a reduced intake of food (usually a 20–40% reduction in habitual caloric intake) while maintaining normal meal frequency or, in some protocols, skipping dinner [2]. CR can be performed continuously or intermittently, such as one or few days per week. The intake of 500–700 kcal on one or more days per week, often with a prevalence of proteins, is considered Semi-Fasting (SF = more than 40% caloric reduction), as in, for instance, the 5:2 diet, that is, 5 days a week with normal nutrition and 2 non-consecutive days with 5-600 kcal (Figure 1).

An emerging approach is the Fasting Mimicking Diet (FMD), typically implemented for five consecutive days, 2 to 10 times per year. On day 1, the intake is approximately 1150 kcal, mostly vegetable-based, with a macronutrient distribution of 43% carbohydrates, 46% fats, and 11% proteins. From days 2 to 5, intake is reduced to ~725 kcal/day, composed of 47% carbohydrates, 44% fats, and 9% proteins [3] (Figure 1).

Time-Restricted Feeding (TRF) is another approach that also gained attention. It involves a daily fasting window of 12–16 h, typically during the nighttime period, not necessarily with a caloric restriction [4] (Figure 1). Notably, religious forms of fasting, such as Ramadan and Sunnah fasting, should not be overlooked [5].

The popularity of semi-fasting and TRF is rapidly increasing and may be largely due to their higher acceptability and adherence rates [6] compared to traditional continuous CR, especially in the context of weight loss interventions.

### 2.1. Fasting and Its Closest Variants: A Modern Trend?

The growing popularity of fasting is driven by the increased public demand for weight loss and general well-being, the speculative pressures of the diet industry, and the increasing amount of scientific evidence supporting the potential benefits of certain degrees of CR in the prevention of metabolic, cardiovascular, oncological, neurodegenerative, autoimmune, and inflammatory diseases, as well as its possible role in promoting longevity.

As of 2024, the World Wide Web hosts about 1 billion websites across all sectors, of which approximately 190–200 million are active and regularly updated. Although precise data on websites dedicated to CR are lacking, they are estimated to be about tens of millions. In addition, thousands of books have been published on this topic [7].

A bibliometric analysis using the keywords “fasting”, “intermittent fasting”, and “time-restricted feeding”, from 2000 to 2025 reveals a substantial increase in related publications indexed in PubMed, such as Fasting from 2218 to 8971 publications (+304%); Intermittent fasting from 7 to 607 publications (+8571%); Time-restricted feeding: from 42 to 807 publications (+1821%).

### 2.2. Fasting Come First

The term fasting derives from the Latin word ieiunum, meaning “on an empty stomach.” Its practice can be traced back as early as 3000 BCE in ancient civilizations, where it was used for spiritual purposes, i.e., for deeper understanding of the human soul, through the purification of both body and spirit, observing the silence, intended as abstinence not only from food but also from verbal and environmental stimuli, and respecting the divine ritual.

The ancient Egyptians reportedly fasted for approximately three days per month, especially during funerary rites. Fasting was also practiced within shamanic and druidic traditions, mystical-initiatory groups (such as the Pythagoreans, Essenes, Sufis, Cathars, Stylites, and anchorites), and across both monotheistic and polytheistic religious systems, including Judaism, Christianity, Islam, and Vedic, Brahmanic, Hindu, Buddhist, Jain, and Yogic traditions.

Over time, fasting became associated with self-discipline, initiation ceremonies, promotion of masculinity, and, more recently, acts of civil protest (1909 Suffragette Movement Great Britain) [8].

If fasting purifies the spirit, why should it not also purify the body? Would it have remained so widespread and enduring if had it not offered some form of physical benefit, beyond its role in facilitating access to the unconscious through dreams, myths, visions, and legends?

From a modern scientific standpoint, we may also ask if those who practiced fasting lived longer lives. The issue remains speculative, although historical anecdotes provide intriguing suggestions. Pharaoh Ramses II (1304–1213 BCE), who also served as a high priest and was plausibly engaged in ritual fasting, is believed to have lived to age 91. Likewise, many of the major philosophers of Ancient Greece, living between 575 BCE and 127 BCE and practicing fasting, were reported to have survived well beyond the average life expectancy of their time, which was about 41 years for those who made it past age 15. In particular, Pythagoras lived 75 years, Socrates 71 years, Plato 80 years, Hippocrates 83 years, and Plutarch 79 years. These historical anecdotes do not provide scientific evidence of a causal relationship between fasting and longevity. Indeed, this longevity could have been merely coincidental or even related to higher intellectual functioning [9].

### 2.3. Fasting: Historical and Ethnographic Notes

Early scientific investigations of fasting date back to 1843, when Charles Chossat examined the effects of prolonged food deprivation in pigeons [10].

Subsequent animal studies further investigated tissue-specific adaptations to CR and showed that different degrees of CR lead to both reduced metabolic rate and decreased body mass, though these effects were unevenly distributed [11].

During the 19th century, fasting was also promoted as a therapeutic practice, although its proposed detoxifying effects lacked scientific validation [12].

In 1889, Italian cardiologist and physiologist Luigi Luciani conducted a scientific study on professional faster Giovanni Succi and published the first formal scientific paper on the subject: Fisiologia del Digiuno [13]. In 1915, American chemist and physiologist Francis Gano Benedict documented a 31-day fast by Maltese naturalist Agostino Levanzin in A Study of Prolonged Fasting. Two years later, the first experimental evidence linking CR to lifespan extension in rodents was published [14].

Historical and ethnographic accounts have associated traditional fasting practices of several populations (e.g., Kitava people of Papua New Guinea, Hunza people of the Himalayan region, and Greek rural communities) with favorable health outcomes and longevity; however, these observations remain anecdotal or observational and do not establish causality [15,16,17].

Later human investigations, including the Minnesota Starvation Study, the Spanish hospice study, and Biosphere 2, contributed to understanding physiological adaptations to energy restriction [18,19,20].

More recent studies of religious fasting, including Ramadan, have reported modest metabolic changes, although findings remain heterogeneous and do not establish long-term clinical benefits [21]. An overview of the main dietary strategies based on fasting and caloric restriction, their target populations, principal outcomes, levels of evidence, and major limitations is provided in Table 1.

## 3. Fasting: Physiological Aspects

In the plant kingdom, fasting is represented by the dormancy during winter, when leaves are shed [22]. In the animal kingdom, hibernation and estivation represent forms of fasting. Moreover, migratory birds and spawning salmon also refrain from eating during their journeys [23]. During severe famine it was observed an energy shift from reproduction and growth toward tissue defense and repair, resulting in decreased DNA synthesis and cellular proliferation [24].

### 3.1. Brain Metabolism

Over 200,000–300,000 years of evolution, periodic food scarcity likely drove the development of the human nervous system [25,26]. The brain is somehow a selfish organ. It represents only ~2% of body mass but consumes ~20% of oxygen. β-hydroxybutyrate is the most efficient fuel for the brain, reflecting evolutionary adaptation favoring fat-burning before the increase of carbohydrate intake that took place during the Neolithic period.

Accordingly, it is now accepted that alternating ketosis and nutritional recovery may optimize cognitive function (Intermittent Metabolic Switching—IMS). 

During ketosis, oxidative stress is reduced, and autophagy and BDNF (Brain Neurotrophic Factor) production are stimulated. Neurons become metabolically conservative and counteract stress. During refeeding, the return of glucose and amino acids, combined with the accumulated high levels of BDNF, triggers synaptogenesis, synaptic plasticity (LTP—Long Term Potentiation), and the growth of new neurons in the hippocampus. Alternation accustoms the brain to metabolic flexibility, i.e., the ability to quickly and effectively switch from consuming sugars to fat/ketones and vice versa [27].

Under energy stress, the expression of insulin-independent GLUT-1 receptors increases [28,29,30]. Preclinical studies suggest that abstaining from food (or exposing oneself to cold) for 24 h is enough to form, especially in the hippocampus, new synaptic connections and to improve neurogenesis, probably due to ketones that increase levels of Brain-Derived Neurotrophic Factor (BDNF) [31]. However, these findings obtained in animal models cannot be interpreted as evidence of neuroprotective effects of fasting in humans.

### 3.2. Tissue Metabolism

Fasting and cold exposure modulate genes related to hunger, longevity, fertility, and immunity. The first phases of semi-fasting (SF) rely on hepatic and muscle glycogenolysis, while lipolysis of adipose tissue starts later. Free fatty acids induce muscle insulin resistance to save glucose for glucose-dependent tissues, eventually triggering a metabolic shift from glucose to ketones. Ketogenesis starts ~12–16 h into fasting or low carbohydrate intake (<50 g/day), with mild ketosis at 2–3 days. In 3–7 days, ketone bodies are consistently utilized, and full adaptation is achieved in about 2–4 weeks. Hepatic ketogenesis supplies ~⅔ of cerebral energy needs, with hepatic and renal gluconeogenesis covering the rest. Muscle, cardiac, hepatic, and renal cells utilize ketones immediately, while neurons require an adaptation period [32].

### 3.3. Evolutionary Adaptations of Energy Efficiency

Ketone bodies provide an alternative mitochondrial fuel and may yield more ATP per unit of substrate mass than glucose [33]. In the first 3–4 days of lowered food intake, protective mechanisms are activated: increased fat storage efficiency, increased resting energy expenditure (REE) of about 4–12%, enhanced muscle strength, fatigue and stress resistance, and improved sensory and cognitive functions [34]. At 12–24 months the REE drops by about 5% with a CR of 25%. Evolution favoured individuals with higher resilience to fasting, especially at the brain level. They have higher surviving and reproducing chances [35].

In a condition of severe food shortages, there is an energetic shift from reproduction and growth to tissue defense and repair, with a reduction of DNA synthesis and an increase in the antiproliferative effect [36].

### 3.4. Clinical Signs & Early Symptoms

During the first 15–20 days of fasting, individuals may experience a wide spectrum of subjective symptoms, such as discomfort, constipation or diarrhea, nausea, halitosis, dry mouth, reduced sweating, headaches, early asthenia, hypoglycemia, and dizziness. Due to waste elimination, it is often observed a white tongue coating, mostly resolving in about 29 days, and malodorous sweating. Body temperature fluctuates slightly, then declines. It is worth noting that individuals with an internal temperature lower than the median typically live longer without CR [37]. Cold sensitivity increases due to catecholamine-induced vasoconstriction. Blood pressure decreases due to reduced sympathetic tone, while the resting heart rate remains stable with possible extrasystoles under stress. Adipose tissue exhibits increased circulation in the attempt to mobilize energy. Gastrointestinal changes include possible pyloric hypertonia, gastric stagnation, and, in the first few days, increased motilin secretion at the duodenal/proximal jejunal level to facilitate the clearance of residual food. Intestinal mucosa thins and peristalsis slows. Libido and urinary flow may decline [38]. During the first 2–3 days, edema does not occur because the amino acids released from the muscles maintain the blood’s oncotic pressure. However, when even a small amount of carbohydrates is present, the osmotic pressure is no longer adequately compensated by amino acids and proteins, leading to early edema. As fasting continues, the production of ketone bodies requires fewer enzymes than carbohydrate metabolism. These ketone bodies remain longer in circulation, helping to maintain oncotic pressure and prevent late-onset edema [24].

Some studies indicate that short-term fasting can increase negative emotions, such as depression, anxiety, anger, irritability, fatigue, and tension, while decreasing positive emotions and vitality [39], while others have found opposite effects [40,41]. This ambivalence is likely related to the degree of personal motivation for fasting.

Recently, a comprehensive review showed no difference in cognitive performance between healthy fasting and non-fasting adults, while negative effects have been found in children [42,43].

### 3.5. Hormesis & Autophagy

**Figure 2:**
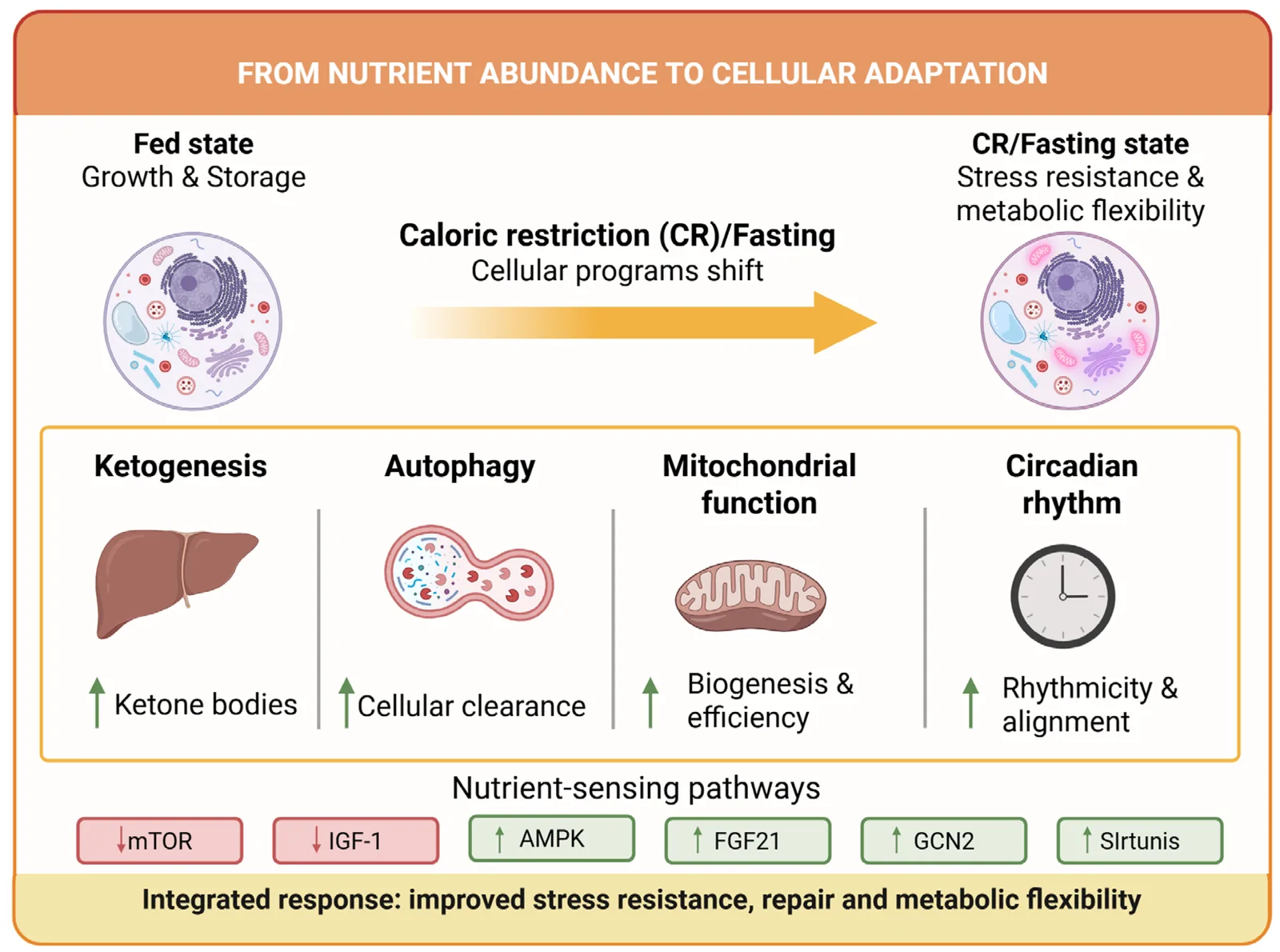
**Cellular Adaptations Induced by Caloric Restriction and Fasting.** Schematic representation of the metabolic and cellular adaptations induced by caloric restriction (CR) and fasting. The transition from a nutrient-abundant fed state, characterized by growth and energy storage, toward a CR/fasting state promotes cellular stress resistance and metabolic flexibility. These adaptations include enhanced ketogenesis, activation of autophagy, improved mitochondrial biogenesis and efficiency, and better circadian rhythm alignment. At the molecular level, CR and fasting modulate nutrient-sensing pathways through the downregulation of mTOR and IGF-1 signaling and the activation of AMPK, FGF21, GCN2, and sirtuins. Collectively, these coordinated responses contribute to improved cellular repair, stress resilience, and metabolic health.

CR turns act as physiological hormetic stressors. The word hormesis derives from the Greek hormao meaning “to stimulate”. Indeed, this phenomenon describes how low doses of a substance act as a beneficial training stimulus for the body, while beyond a certain cut-off point, it produces the opposite effect. As an example, hormetins present in Mediterranean foods (e.g., allicin, resveratrol, curcumin, sulforaphane, and capsaicin) are able to activate the Nrf2–ARE (Nuclear Factor Erythroid 2-related factor 2-gene transcription factor; ARE-Antioxidant Response Element) that allows the activation of anti-antioxidant and anti-inflammatory cellular pathways, promoting mitochondrial biogenesis [44]. Cellular turnover averages about 52 days across 250 human cell types. Fasting and ≥25% CR potentiate autophagy, including ribophagy, mitophagy, peroxisome- and ER-phagy, eliminating, degrading, and recycling damaged components. Mitochondria, the powerhouse of the cell, wear out quickly and need to be renewed through mitochondrial biogenesis, which increases with CR [32,45]. This increase is mainly driven by nutrient-sensing pathways activated during energy scarcity. In particular, caloric restriction raises the AMP/ATP and NAD/NADH ratios, leading to the activation of AMPK and SIRT1, which converge on PGC-1, the master regulator of mitochondrial biogenesis [46] (Figure 2). Once activated, PGC-1 coactivates transcription factors such as NRF1 and NRF2 and promotes the expression of TFAM, thereby enhancing mitochondrial DNA replication, transcription, and the synthesis of mitochondrial proteins [47] (Figure 2). At the same time, the reduction in insulin/IGF-1 and mTOR signaling shifts the cell from an anabolic to a maintenance-oriented state, favoring mitochondrial turnover and quality control [47] (Figure 2). In this context, mitochondrial biogenesis works in concert with mitophagy, ensuring that damaged mitochondria are removed while new and functionally efficient organelles are generated, ultimately preserving cellular energetics and metabolic flexibility during nutrient limitation. Lysosomes, which are nutrient sensors, potentiate their activity with fasting, reducing inflammation and facilitating waste clearance [48] (Figure 2).

## 4. Interaction Between Circadian Clocks, Feeding Behavior, and Metabolism

### 4.1. Circadian Clocks and Feeding Behaviour

Nowadays, particularly in clinical practice, still limited attention is paid to circadian rhythms, despite their central role in the regulation of feeding behavior and metabolism. Food intake is not driven exclusively by an actual energy deficit but rather by anticipatory mechanisms aimed at preventing its occurrence, and this is an evolutionary adaptation that has favored survival. For instance, the metabolism of a nocturnal animal is normally active during nighttime. If the animal was wrongly looking for food during daytime hours, it would display increased vulnerability to predators [49].

The circadian system in humans is organized around a central clock, located in the suprachiasmatic nucleus of the hypothalamus, and multiple peripheral clocks, present in the adrenal glands, heart, intestine, adipose tissue, thyroid, pancreas, liver, and skeletal muscle, and at the molecular level also within each of about 37 trillion human cells. These peripheral clocks may become desynchronized from the central pacemaker in response to changes in meal timing [50].

Studies in animal models demonstrate that subjects fed during their resting phase (daytime for nocturnal animals) gain weight, compared to control animals, despite equivalent caloric intake. Moreover, they develop food anticipatory behavior, learning to awaken approximately one hour before food availability and subsequently returning to sleep. If this feeding pattern persists, a reset of the hepatic clock and of metabolic and digestive clocks is observed, with a shift from night to day, occurring partially independently of central brain control [51].

The suprachiasmatic nucleus is directly connected to hypothalamic hunger centers and is responsible for the temporal regulation of feeding drive. Following circadian rhythms, the intestine is able to produce digestive enzymes and secretions shortly before food intake, thereby optimizing digestive efficiency. Although a certain degree of adaptation is possible, chronic misalignment with natural biological timing ultimately leads to metabolic dysfunctions [52].

Nutrient absorption is a metabolically demanding process that cannot remain continuously active, but it is more efficient under conditions of physical activity and natural light exposure, both key cues for circadian synchronization. Exposure to early morning light represents the primary resetting signal for the central clock, whereas the first meal of the day synchronizes peripheral clocks. Thus, meal timing constitutes a powerful modulator capable of overriding or attenuating signals originating from the central nervous system. Sleep deprivation for even a single night reduces the responsiveness of brain regions involved in food-related decision-making while increasing activation of reward-related areas, negatively influencing dietary choices [53].

Insulin sensitivity is highest in the morning hours, with more efficient carbohydrate metabolism during the first half of the day, inversely correlated with melatonin levels. The immune system is also regulated according to a precise temporal organization: processes of immune surveillance, inflammatory response, tissue repair, and clearance of cellular debris occur at distinct times of the day, mainly at night, to prevent excessive physiological burden. In this context, skeletal muscle tissue exhibits greater capacity for nutrient uptake and utilization during the late day and nighttime hours, when recovery and structural regeneration processes are more active [54].

Experiments with murine models demonstrated that a healthy diet consumed within a restricted feeding window of 8–10 h resulted in increased muscle mass compared with unrestricted feeding and the ability to reset biological clocks under conditions of circadian desynchronization [55]. Furthermore, tumor-bearing mice subjected to time-restricted feeding (TRF) exhibited a significant slowing of tumor growth [56].

Disruption of circadian oscillations through genetic manipulation of clock genes (such as *Clock*, *Per1*, and *Cry*) induces obesity, alters glucose metabolism, and reduces lifespan in murine models [57]. Similarly, irregular feeding patterns uncouple peripheral clocks from the hypothalamic central pacemaker, promoting insulin resistance and glucose intolerance [58].

The gut microbiota is also profoundly influenced by circadian rhythms, exhibiting daily fluctuations in composition. Animal models fed high-fat and high-carbohydrate diets display reduced microbial diversity and an increased propensity toward obesity [59]. A prolonged fasting period may reduce intestinal permeability, increasing the risk of endotoxemia, i.e., presence of endotoxins in the bloodstream, upon refeeding. After one month of intermittent fasting, an increase in *Akkermansia muciniphila*, a marker of metabolic health, as well as in *Parabacteroides distasonis* and *Bacteroides thetaiotaomicron*, has been observed [60].

### 4.2. How Long Can Humans Survive While Fasting?

Under average conditions, survival of human beings without water intake is limited to approximately 7–10 days, whereas survival without food, provided that adequate hydration is maintained, may extend to roughly 40–80 days, with considerable interindividual variability. However, irreversible physiological damage may already occur after 15–30 days of prolonged fasting, depending on baseline body reserves, initial nutritional status, and underlying clinical conditions [61].

The mortality threshold is generally associated with a body mass index below 13 kg/m^2^ or a body weight loss exceeding 40–50% of initial body weight, conditions that reflect a critical depletion of the organism’s energetic and structural reserves.

### 4.3. Fasting and Changes of Hematochemical Parameters

(1). Glycemic homeostasis and substrate selection

After the first 7–8 days of fasting, blood glucose tends to stabilize around 55–60 mg/dL, reflecting the metabolic priority assigned to carbohydrates over lipids as an energy substrate for glucose-dependent tissues. When availability of glucose to adipocytes is reduced, long-chain fatty acids are released and preferentially oxidized by skeletal muscle, thereby sparing glucose for other organs, in accordance with the Randle cycle. In this context, alternate-day fasting (ADF) appears to improve rates of insulin-mediated glucose uptake [62].

(2). Ketogenesis and cerebral utilization of ketone bodies

From approximately the fourth day of fasting, the brain, for about 75% of its energy requirements, uses ketone bodies, particularly β-hydroxybutyrate, whose utilization increases from ~50 to 100 g/day. The preferential use of ketone reduces pyruvate entry into the Krebs cycle, thereby decreasing protein-derived gluconeogenesis and, consequently, limiting the loss of structural and essential proteins. Ketone bodies may also exert detoxifying and antioxidant actions [63]. In adult humans, ketone body production after an overnight fast is negligible; plasma β-hydroxybutyrate levels begin to rise after 18–24 h of fasting and increase progressively (more rapidly in women than in men), reaching 4–7 mM after approximately two weeks of fasting [64]. During fasting, β-hydroxybutyrate also acts as a signaling molecule by binding the G protein–coupled receptors GPR109A and GPR41. This interaction inhibits histone deacetylase, inducing broad changes in gene expression and signaling pathways involved in protection from oxidative stress. In rodents, ADF also exerts neuroprotective effects by increasing concentrations of BDNF [28]. However, other evidence has reported a marked reduction in circulating BDNF levels, highlighting that metabolic adaptations to ADF in mammals with very high metabolic rates (e.g., young rodents) may not be directly extrapolated to middle-aged or older adults [65]. Thus, the clinical relevance of these findings in humans remains uncertain.

(3). Total body nitrogen content, averaging approximately 1 kg, progressively declines during fasting and reaches a critical (vital) threshold of 50% after at least 3–4 weeks [66]. Within the first 5 days, a marked decline in gluconeogenic amino acids, particularly alanine and glutamine, is observed, consistent with activation of renal gluconeogenesis.
(4). Electrolyte and mineral variations are generally modest; however, after several weeks, plasma potassium may approach 3 mEq/L [67].
(5). Total cholesterol may fluctuate in either direction, whereas triglycerides and transaminases tend to increase, likely reflecting increased hepatic turnover.
(6). Blood pH remains stable during the early phases of fasting due to increased aciduria, acceptable ketonemia levels, and buffering supported by alkaline molecules derived from autophagy. A similar effect has been observed in the human brain [68].
(7). During the first 10 days of fasting in humans, uric acid levels rise, likely due to changes in renal function [69,70]. Uric acid appears to exert a hormetic effect—acting as an antioxidant in a hydrophilic environment at concentrations below 3–5 mg/dL but promoting mitochondrial free radical generation at higher levels [71]. Under oxidative stress conditions, it can neutralize more than 60% of free radicals. If increases in uric acid were purely harmful, it would be difficult to explain the evolutionary advantage of urate oxidase (uricase) gene silencing—absent only in humans and great apes—as well as the fact that the kidneys reabsorb more than 90% of filtered urate via URAT-1 and GLUT9 transporters [72].

### 4.4. Fasting and Endocrine Responses

From a hormonal standpoint, insulin decreases during the first 5 days, followed by stabilization [73]. Glucagon rises during the first 2–3 days, then stabilizes at slightly above-normal levels. Norepinephrine increases progressively with a peak around 20 days, whereas epinephrine shows a mild increase after approximately day 15, likely as an attempt to counteract metabolic downregulation and the stress associated with caloric restriction. Aldosterone levels remain largely stable, whereas cortisol increases. The glucocorticoid receptor GR-α is inhibited, while the GRβ isoform, induced by TNF-α and IL-1, antagonizes GR-α, resulting in an attenuated immune/inflammatory response and favoring the development of hyperinsulinemia.

In premenopausal women undergoing a 5:2 dietary regimen for 24 weeks, no significant changes were observed in testosterone, androstenedione, DHEA-S, SHBG, or prolactin. By contrast, in young men undergoing 8-h TRE for 8 weeks, reductions in total and free testosterone were reported [74]. These findings suggest that endocrine responses may vary according to sex and dietary protocol. However, due to the differences in study populations, duration of intervention, and energy restriction paradigm, no consistent sex-specific response can be established.

Adiponectin increases after the first 72 h of fasting, exerting an overall effect that may counteract pre-existing inflammation [75]. TNF-α and T3 show an initial increase followed by a decline; IL-6 and homocysteine decrease. Leptin declines sharply, likely activating a homeostatic feedback that suppresses reproductive function, thyroid hormone secretion, and energy expenditure, suggesting a primary role in energy conservation rather than in preventing excessive fat accumulation [76]. Ghrelin follows a similar but pulsatile pattern, with lower levels during sleep unless danger is present [77].

Circulating growth hormone (GH) levels tend to approximately double after about 4 days of fasting due to loss of glucose- and lipid-mediated suppression [78]. The GH/IGF-1 axis represents a central metabolic node involving the hypothalamus, pituitary, and liver, and is strongly modulated by nutrient availability and insulin. GH and IGF-1 stimulate protein synthesis and bone development but exert opposing effects on lipolysis and insulin sensitivity. IGF-1 levels, which remain stable throughout the day, provide a useful marker of average GH exposure.

IGF-1 exerts both beneficial and potentially adverse effects: it supports anabolism, cardiovascular health, and nutritional status, yet also promotes cellular proliferation, thereby increasing neoplastic risk [79]. In the brain, IGF-1 promotes neurogenesis, synaptogenesis, and myelination but may also increase free radical production and inhibit autophagy, interfering with stress resistance and cognitive processes [80]. In neurodegenerative disorders characterized by protein aggregation, reduced IGF-1 stimulation may slow progression and improve protein clearance.

During fasting in humans, a reduction in the number of somatotropic receptors contributes to declining circulating IGF-1 levels. In Laron syndrome, high GH levels combined with low IGF-1 correlate with reduced cancer and diabetes risk, without a clear increase in longevity. IGF-1 contributes to telomere length maintenance and telomerase activity in several animal species, but not in humans; it may nevertheless play an indirect role through effects on stress and mood [81,82].

Fasting transiently activates the endocannabinoid system to increase the intake of energy substrates (hunger stimulus, increased sensitivity to ghrelin, and increased sweet taste). The antinausea, anti-inflammatory, and anti-emetic effect is an evolutionary response to the risk of side effects due to the intake of new foods [83,84].

### 4.5. Sex-Specific Responses to Fasting and Caloric Restriction

Sex-related differences in energy metabolism and endocrine regulation may influence the physiological response to fasting and CR. In females, both rodents and humans, during starvation or prolonged exercise, estrogens allow them to rely on lipid oxidation as an efficient energy source, preserving glucose for neuronal functions and proteins for other organ functions [85]. In general, women exhibit higher circulating ketone levels than men during fasting, which may reflect differences in fatty acid availability, substrate utilization, and hormonal regulation [86].

Reproductive endocrine function is another potentially important source of sex-related variability: in women, responses may be influenced by menstrual status, ovarian hormone fluctuations, and baseline energy availability; in men, changes in testosterone have been reported in some intermittent fasting studies [87]. Nevertheless, human evidence remains limited, and significant gaps are present in the literature. For instance, in a 12-month randomized trial involving adults with obesity, it was not observed any significant alterations in circulating testosterone, DHEA, or sex hormone-binding globulin (SHBG) among men, premenopausal women, or postmenopausal women following time-restricted eating or daily CR [88]. On the other hand, prolonged or severe energy restriction and inadequate energy availability may affect reproductive function [89]. Therefore, further controlled studies incorporating sex-stratified analyses of relevant reproductive and metabolic outcomes are needed to clarify these differences and to design personalized dietary recommendations.

## 5. Molecular Pathways Involved in Caloric Restriction, Fasting, and Semi-Fasting

CR, through its diverse quantitative and temporal patterns, activates a complex and highly integrated network of signaling pathways that regulate nutrient sensing, energy metabolism, cellular growth, and the induction of maintenance and repair mechanisms. The reduced availability of carbohydrates and specific amino acids, such as methionine and branched-chain amino acids (BCAAs), is detected at the cellular level as a state of metabolic stress. This condition triggers a broad functional reprogramming of tissues aimed at optimizing energy utilization and enhancing cellular resilience. One of the earliest molecular events triggered by CR is the activation of AMP-activated protein kinase (AMPK), a key cellular energy sensor modulated by ATP/AMP ratio [24]. In parallel, CR suppresses proliferative signaling through the mammalian target of rapamycin complex 1 (mTORC1), primarily via activation of the tuberous sclerosis complex (TSC) and the Rag–GATOR1 (Gap Activity Toward Rags 1) pathways [90]. These regulatory systems control the recruitment of mTORC1 to the lysosomal surface, a crucial step for its activation and, consequently, for the regulation of cellular growth and metabolism.

mTOR is the catalytic core of two distinct multiprotein complexes, mTORC1 and mTORC2, each characterized by specific subunits and biological functions. mTORC1, which is sensitive to rapamycin, integrates environmental signals such as the availability of amino acids, glucose, oxygen, cholesterol, and insulin/IGF-1, thereby regulating protein synthesis, ribosomal biogenesis, and cell proliferation. In contrast, mTORC2 is less directly responsive to nutrient availability and mainly functions as an effector of phosphoinositide 3-kinase (PI3K) signaling, playing a central role in cell survival and cytoskeletal organization [91]. A ~30% level of CR is sufficient to downregulate the insulin–IGF-1–FOXO axis and the PI3K–AKT–mTOR cascade at both transcriptional and post-transcriptional levels. This drives a reprogramming of organs and tissues, shifting from a growth- and proliferation-oriented state toward maintenance, repair, and cellular survival [92]. When mTORC1 signaling is reduced, ribosomal biogenesis and global protein synthesis are suppressed, while autophagy is enhanced. Reduced availability of proteins and amino acids is sensed not only by the Rag–GATOR machinery but also by the amino acid deprivation pathway mediated by General Control Non-derepressible 2 (GCN2), an evolutionarily conserved serine/threonine kinase functioning as a key amino acid sensor [93].

GCN2 coordinates several nutrient-responsive processes, including immune system adaptation [94], tumor cell survival and proliferation under nutrient deprivation [95], and regulation of integrated stress responses and inflammasome activity in the gut [96]. GCN2 activation leads to phosphorylation of eukaryotic initiation factor 2α (eIF2α), causing a transient suppression of global translation and selective induction of Activating Transcription Factor 4 (ATF4) [96]. ATF4 promotes the expression of genes required for adaptation to amino acid and protein restriction and stimulates hepatic production of fibroblast growth factor 21 (FGF21), an endocrine mediator that plays a central role in the systemic response to protein restriction. FGF21 promotes fatty acid oxidation and lipid catabolism and stimulates insulin-independent glucose uptake in both animal models and humans [97]. At the cellular level, FGF21 induces activation of peroxisome proliferator–activated receptor gamma coactivator-1α (PGC-1α) via NAD^+^-dependent Sirtuin-1 (SIRT1), thereby enhancing mitochondrial respiratory activity.

Murine models overexpressing FGF21 is resistant to excess weight gain [98]. FGF21 also exerts central effects in hypothalamic circuits, mediating endocrine control of simple sugar intake and modulating feeding behavior. In particular, elevated FGF21 signaling in the paraventricular nucleus of the hypothalamus is associated with reduced sugar-seeking behavior and greater protection against age-related metabolic alterations [99]. Reduced carbohydrate and overall energy availability decreases IGF-1 signaling, leading to attenuation of phosphoinositide 3-kinase (PI3K) activity and, indirectly, of mTORC2 signaling. Under physiological conditions, activation of AKT inhibits gene expression mediated by the protein FOXO (Forkhead box O), involved in oxidative stress resistance, longevity pathways, and cell growth control. During calorie restriction, reduced AKT signaling favors FOXO-dependent transcriptional programs and contributes to the downregulation of mTORC1 activity. Methionine restriction decreases levels of S-adenosylmethionine (SAM), thereby reducing DNA and histone methylation, highlighting a direct dietary influence on the epigenome. CR also increases expression of sirtuins, a family of NAD^+^-dependent deacetylases encoded by SIRT genes (SIRT1–SIRT7), characterized by distinct subcellular localizations and functions. SIRT1, SIRT6, and SIRT7 are predominantly nuclear, while SIRT3, SIRT4, and SIRT5 are mitochondrial, and SIRT2 is largely cytoplasmic but shuttles between cytoplasm and nucleus [100]. Sirtuins regulate key processes, including aging, transcriptional control, apoptosis, oxidative stress resistance, modulation of inflammation, and energy efficiency, and are preferentially activated in energy deficit states. At the central level, SIRT1 promotes adaptive responses to dietary restriction through activation of dorsomedial and lateral hypothalamic nuclei involved in energy balance and feeding behavior [101]. During fasting, sirtuin-dependent programs contribute to systemic “maintenance” responses, supporting repair mechanisms of damaged cells. These programs are further potentiated by other hormetic stimuli such as cold exposure, physical activity, and polyphenol intake [24,102]. CR induces SIRT1 expression across multiple tissues, notably in human skeletal muscle, where it associates with increased mitochondrial biogenesis [103]. Conversely, overnutrition and obesity are associated with reduced SIRT1 expression in adipose tissue, and similar reductions have been observed in Alzheimer’s disease [104,105]. Key metabolic effects of sirtuins are mediated by activation and nuclear localization of PGC-1α, a master regulator of mitochondrial biogenesis, and metabolic adaptation to exercise, fasting (hepatic gluconeogenesis and fatty acid oxidation), and cold (activation of uncoupling protein 1) [106].

Fasting and semi-fasting reduce circulating IGF-1 and transforming growth factor-β (TGFβ) while enhancing antioxidant defenses, mitochondrial function, proteostasis, and autophagy [107,108,109,110].

In human skeletal muscle, long-term CR upregulates protein quality control systems, increasing molecular chaperone activity (HSP70) for protein folding and glucose-regulated protein GRP78, crucial for endoplasmic reticulum function. In parallel, it increases the autophagy-related proteins LC3 and beclin-1, both required for autophagosome formation, and enhances expression of genes involved in mitochondrial biogenesis via PGC-1α [92,111].

Another signaling system involved in fasting is the family of peroxisome proliferator-activated receptors (PPARs). These nuclear receptors act as transcription factors regulating genes involved in lipid and glucose metabolism as well as inflammatory responses. During fasting, activation of PPARs in key metabolic organs promotes fatty-acid β-oxidation, improves lipid handling, stimulates the biosynthesis of polyunsaturated fatty acids, exerts anorexigenic effects, and modulates central dopaminergic pathways involved in energy balance and reward [112].

Overall, CR activates coordinated cellular repair and recycling programs, including autophagy, mitophagy, DNA repair, and antioxidant defenses, as well as improving adult stem-cell function. As a consequence, cellular senescence is attenuated and proteostasis is enhanced [113].

## 6. Caloric Restriction and Fasting Effects on Metabolic Health, Aging, and Longevity

The molecular mechanisms by which CR promotes health and longevity remained obscure until the 1980s, when the first gene mutations influencing nutrient-sensing pathways were identified in worms and nematodes [114]. CR has been known for nearly a century for its ability to extend lifespan and delay the onset of age-associated diseases in animal models [115]. However, the extent of these effects and the underlying mechanisms vary across species and experimental conditions. Therefore, it is unclear whether these effects can be translated to humans. In mice ‘aged’ brains, the activity of genes associated with inflammation, immunity, and endothelial function of brain vessels increases, while that of genes related to neuronal function and structure decreases. The hypothalamus is a crucial brain region in this switch. The most significant changes in gene expression were found in cell types near the third ventricle of the hypothalamus, including tanicytes and ependymal cells, and in neurons known for their role in food intake, energy homeostasis, and metabolism [116].

Multiple factors contribute to the regulation of aging and age-related pathologies, including genetic, epigenetic, microbial, and molecular determinants. From an epigenetic perspective, CR is associated with DNA hypermethylation and gene silencing. At the molecular level, the key signaling pathways involved include AKT–FOXO–mTOR, FGF21, GCN2–ATF4, AMPK, and sirtuins [117]. It is interesting that reduced insulin signaling and attenuation of the IGF1 axis represent fundamental determinants of lifespan extension, with decreased mTOR activity acting as a convergent mechanism of multiple endocrine longevity-promoting factors [118].

Several human studies suggest that CR significantly improves metabolic health. These findings support the hypothesis that fasting-mimicking dietary regimens may activate regenerative programs, including stem cell–based mechanisms, through the transient inhibition of major anabolic pathways. Consistently, prolonged fasting reduces IGF1/PKA signaling, thereby promoting hematopoietic stem cell–based regeneration and reversing immunosuppression [119].

Long-term studies in *Rhesus Macaques* subjected to a 30% CR have yielded partially discrepant results. One study indicated about a 50% reduction in mortality compared with controls [120], while another one reported improvement in health span without a significant extension of lifespan [121]. In both cases, however, CR markedly delayed age-associated DNA methylation, resulting in an estimated biological age approximately seven years younger than chronological age [122]. These non-human primate studies provide important translational evidence, emphasizing the difference between improving health span and extending lifespan. Anyway, given these results from animal models, it is not possible to generalize the findings to humans.

In murine models, a 24-h fast enhances intestinal stem cell functions through the induction of fatty acid oxidation, mediated by increased activity of carnitine palmitoyltransferase 1 (CPT1), the rate-limiting enzyme of β-oxidation, secondary to reduced malonyl-CoA levels [123]. 

In murine models, protein restriction exerts effects on longevity, particularly in males, independently of total caloric intake, in contrast to CR, which is effective in both sexes [124]. Conversely, a high protein intake (>1.3 g/kg/day) has been associated with insulin resistance and type 2 diabetes mellitus in human observational studies, hyperactivation of the AKT–mTOR axis, inhibition of the GCN2–ATF4 pathway, and consequent reduction of FGF21 levels in both murine models and in vitro studies [125]. In this context, excessive protein intake may abolish the metabolic benefits of weight loss on skeletal muscle insulin sensitivity and amplify pro-aging and pro-oncogenic signaling driven by compensatory hyperinsulinemia, despite reductions in visceral adiposity. The underlying mechanisms remain incompletely understood and may involve alterations in muscle fiber structure and increased oxidative stress [126].

The importance of avoiding excessive protein intake is supported by a human epidemiological observational study showing that Okinawan centenarians traditionally consume a diet in which only 9% of total energy derives from protein [127]. However, for the prevention of sarcopenia, particularly after the age of 65, protein intakes exceeding 1 g/kg/day are recommended, as shown by Rondanelli et al. in a human randomized controlled trial study, highlighting the need to modulate protein intake according to different stages of life [128].

In rodents in vitro and in vivo experiments, restriction of individual amino acids has emerged as an additional longevity modulator. Methionine restriction extends lifespan in mice by reducing protein synthesis and increasing FGF21 levels; it also affects hydrogen sulfide (H_2_S) production and S-adenosylmethionine (SAM) availability, resulting in modifications of DNA and histone methylation [129,130]. Similar to protein restriction, methionine restriction increases energy expenditure by promoting browning of white adipose tissue [131]. However, prolonged restriction may induce oxidative stress due to glutathione depletion, an effect that can be counteracted by cysteine supplementation [132,133].

In humans, elevated circulating levels of branched-chain amino acids (BCAAs), particularly isoleucine, are associated with obesity and type 2 diabetes mellitus. In murine models, BCAA restriction reduces obesity, improves insulin sensitivity, and increases energy expenditure but does not elevate FGF21 levels and does not extend lifespan [134,135,136].

Studies in vitro, as well as in animal models in vivo and in humans, showed that restriction of tryptophan, a precursor of NAD^+^ and an essential cofactor for sirtuins, increases FGF21 secretion and negatively correlates with type 2 diabetes mellitus and cognitive performance [137,138]. Similarly, threonine restriction enhances insulin sensitivity and energy expenditure through FGF21-dependent mechanisms [139].

The question of why severe and prolonged caloric reduction extends lifespan across many species remains open. Among candidate molecules, lithocholic acid has been identified as a key mediator of CR-induced anti-aging effects in nematodes, Drosophila, and mice, mimicking AMPK and sirtuin activation through binding to TULP3. Although direct evidence in humans is lacking and high doses may be toxic, elevated circulating levels of lithocholic acid have also been observed in Japanese centenarians [140,141].

With aging, it has been observed an increased activation of catabolic pathways such as the ubiquitin–proteasome system and the production of cytokines, including myostatin and TGF-β, which contribute to muscular and cerebral atrophy. In animal models, metabolic and physical decline with aging appears to be genetically mediated and, in skeletal muscle, is associated with activation of DNA-dependent protein kinase (DNA-PK), a pro-inflammatory kinase that inhibits AMPK and impairs mitochondrial biogenesis.

In summary, several major aging-related processes appear to be affected by CR, including lipid, protein, and DNA oxidation; chronic inflammation; accumulation of dysfunctional proteins and organelles; hyperglycemia; hyperinsulinemia; elevated IGF1 levels; mTOR activity; altered autophagy; and ketogenesis [142,143].

Overall, intermittent fasting appears more advantageous than ketogenic diets, which involve high consumption of animal fats associated with increased levels of trimethylamine N-oxide, a metabolite linked to cardiovascular risk [143,144].

It is noteworthy that the metabolic effects of ketones on the brain appear to be age-dependent: modest in young adults, more pronounced in midlife (40–59 years), when the brain is metabolically vulnerable, but still resilient, and reduced again in older individuals [145]. The available evidence supporting the specific mechanistic effects and potential long-term outcomes of fasting and caloric restriction, together with the main limitations and translational gaps, is summarized in Table 2.

## 7. Recommendations for Specific Clinical Populations

Fasting and CR should not be prescribed as generic dietary strategies. Their safety and clinical value depend on the patient’s metabolic status, medications, kidney function, nutritional reserves, and physical activity.

### 7.1. Type 2 Diabetes

In adults with type 2 diabetes, IF, TRE, and periodic fasting-mimicking diets may be considered alternatives to continuous CR, particularly when weight loss is a treatment goal. However, current evidence supports their use mainly as structured, supervised dietary patterns rather than as inherently superior therapies. In the EARLY randomized clinical trial, a 5:2 meal-replacement strategy improved glycemic control over 16 weeks compared with metformin or empagliflozin, although the intervention was short-term and required careful dietary organization [146].

Patients treated only with lifestyle measures or metformin may be suitable candidates for a moderate TRE schedule, such as a consistent 10–12-h eating window, provided that total energy and nutrient intake remain adequate. More rigid regimens, including alternate-day fasting, very-low-energy fasting days, or fasting-mimicking diets, should be introduced only with professional supervision.

Particular caution is required with insulin and insulin secretagogues because fasting can precipitate hypoglycemia. In insulin-treated T2D, IF has been shown to be feasible when accompanied by medication adjustment and glucose monitoring, but it should not be undertaken independently [147]. Before initiation, clinicians should review insulin or sulfonylurea doses, self-monitoring or continuous glucose-monitoring plans, and hydration and give clear rules for terminating the fast.

### 7.2. Chronic Kidney Disease

Evidence for fasting in chronic kidney disease (CKD) remains limited and is derived predominantly from Ramadan-fasting studies. Stable patients with mild-to-moderate CKD may sometimes fast safely after individualized assessment, but fasting should not be considered routinely safe across all CKD stages [148].

A pre-intervention assessment should include estimated glomerular filtration rate, albuminuria or proteinuria, blood pressure, electrolyte status, volume status, diabetes control, medication schedule, transplant status, and previous fasting tolerance. Patients with unstable kidney function, recent acute kidney injury, significant electrolyte abnormalities, poorly controlled diabetes, advanced CKD, or high fluid requirements should generally avoid fasting.

Patients receiving hemodialysis or peritoneal dialysis should not be encouraged to fast routinely. If a patient nevertheless chooses to fast, the decision requires the nephrologist’s involvement, individualized adjustment of dialysis and medication schedules, and close monitoring of weight, blood pressure, electrolytes, and volume status. Dialysis patients are considered high-risk because fasting can disrupt fluid, potassium, phosphate, and medication management [149].

### 7.3. Older Adults

In mechanistic and preclinical studies, amino acid restriction and modulation of nutrient-sensing pathways, including mTOR and FGF21 signaling, have shown potential benefits for healthy aging. Nonetheless, their application to older adults remains uncertain. Severe fasting, CR, or protein restriction may increase the risk of skeletal muscle and bone loss, functional decline, and reduced resilience. Therefore, weight loss should be recommended selectively, particularly for older adults with obesity-related complications, and avoided as a routine goal for those who are frail, underweight, sarcopenic, or experiencing unintentional weight loss.

When CR is clinically appropriate, a moderate energy deficit (approximately 10–15%), combined with adequate protein intake and resistance exercise, may be more effective in preserving lean mass than severe restriction [150]. Protein intake should be tailored to the individual according to their nutritional status, level of physical activity, kidney function, and other clinical factors. For many older adults, an intake of approximately 1.0–1.2 g/kg/day may be appropriate, although individual requirements can vary. Protein should be distributed across meals, and resistance exercise should accompany CR to help preserve muscle mass and physical function.

TRE may be considered for metabolically stable older adults only when adequate energy, protein, hydration, and safe medication intake can be maintained. Overall, the potential longevity benefits associated with amino acid restriction should not be interpreted as support for routine protein restriction in older adults, for whom adequate protein intake and the preservation of muscle mass and function are essential.

### 7.4. Athletes

For athletes, fasting and CR must be evaluated based on training demands, competition calendars, sex-specific physiology, and the risk of low energy availability. The primary recommendation is to protect energy availability and training quality. Athletes should avoid fasting before high-intensity, prolonged, or technically demanding sessions unless the strategy has been tested as safe during low-priority training. Carbohydrate and protein should be timed around key sessions, and post-exercise nutrition should not be omitted merely to extend the fasting window. IF may help some athletes reduce body fat without obvious short-term reductions in performance, but the evidence is heterogeneous and does not establish fasting as more effective than conventional meal timing [151].

Weight-loss phases should be gradual and supervised by a sports dietitian. Large deficits, repeated fasting days, or combining fasting with high training loads can increase the risk of low energy availability and relative energy deficiency in sport that may impair reproductive, endocrine, immune, bone, cardiovascular, and musculoskeletal health and can reduce performance [152].

### 7.5. Eating Disorders

Fasting and intentional caloric restriction should generally be avoided in people with a diagnosed eating disorder or with clinically significant eating disorder symptoms. In anorexia nervosa, restriction is a core component of the illness; in bulimia nervosa, fasting may function as a compensatory behavior after binge eating; and in binge-eating disorder, rigid dietary rules and prolonged food deprivation may contribute to loss of control over eating [153]. Accordingly, intermittent fasting should not be presented as a neutral weight-management strategy in these populations.

The available literature suggests that intermittent fasting may overlap with eating-disorder psychopathology, including preoccupation with food, guilt after eating, perceived loss of control, and compensatory restriction. In a study of low-carbohydrate dieters, the combination of low-carbohydrate dieting and intermittent fasting was associated with more intense food cravings and disordered-eating features, including binge-eating symptoms and thoughts about food [154]. These findings do not establish that fasting causes an eating disorder, but they support caution when fasting increases dietary rigidity or reinforces weight- and shape-related fears.

During active treatment, the nutritional objective should be restoration of regular, adequate, and flexible eating rather than achievement of a fasting window or calorie deficit. For patients with anorexia nervosa or restrictive eating disorders, treatment may require nutritional rehabilitation, medical monitoring, and gradual normalization of energy intake. For bulimia nervosa and binge-eating disorder, regular meals and planned snacks are commonly used to reduce excessive hunger and interrupt the restriction–binge cycle; treatment should be integrated with eating-disorder-focused cognitive-behavioral or interpersonal therapy [155].

## 8. Conclusions

CR may function as a mechanism of cellular “cleansing” and tissue renewal. However, biological adaptation to fasting is highly heterogeneous, varying not only across species but also among individuals [156]. Inter-individual variability is influenced by multiple factors, including genetic background, age, sex, body composition, metabolic health, lifestyle, circadian rhythms, gut microbiota composition, and environmental exposures. This biological heterogeneity underscores the need to personalize the nutritional recommendations and to use caution against the direct translation of findings from preclinical models to humans. The effectiveness of CR correlates with the variability and complexity of the individuals. For example, although whole-body glycogen content typically averages around 600 g, individual storage capacities vary significantly based on muscle mass, training status, and nutritional state. It is noteworthy that the liver generally stores about 100 to 120 g of glycogen, whereas skeletal muscle can store from 300 g to upwards of 700 g depending on dietary carbohydrate loading. Physiological and biochemical differences, such as training status, dietary habits, genetics, muscle mass, and metabolic phenotype, drive wide fluctuations in whole-body substrate storage and utilization [157]. Moreover, the activation of the same molecular pathway in different individuals can lead to different biological responses, further emphasizing the variability of CR responses [158].

As a consequence, the decision to adopt fasting or semi-fasting practices has to consider the complex biological, behavioral, and psychological individual response. Additional complexity arises from structural limitations inherent to nutritional epidemiology, including the difficulty of accurately measuring caloric intake and the individual variability in dietary patterns [159]. Within this complex biological network, meal frequency and timing appear to play a relevant role.

In overweight individuals, some evidence suggests beneficial effects of time-restricted eating (TRE) protocols with eating windows of approximately 8–12 h, although the magnitude of these benefits is often clinically limited [160,161,162]. Conversely, restricting the eating window to less than 8 h does not appear to confer additional longevity benefits and may even be associated with increased cardiovascular risk [163]. Consequently, the long-term efficacy and safety of these dietary strategies remain uncertain [164]. Based on the currently available evidence, a prudent approach may involve three daily meals distributed within a time window that allows at least 12 h of fasting, with a greater proportion of caloric intake concentrated earlier in the day [165]. However, direct comparisons between early and late TRE remain relatively limited, and findings are not entirely consistent because differences in energy intake, intervention duration, adherence, and participant characteristics may influence the observed outcomes.

In addition, the interval between meals should not be shorter than approximately 4.5 h, as both meal skipping and excessively short inter-meal intervals have been associated with increased all-cause and cardiovascular mortality [166].

People who habitually eat within an 8-h window each day appear to have a higher risk of cardiovascular mortality in observational analyses, compared with those eating across a window of about 11–12 h [167]. The clinical effects of time-qualified dietary interventions are heterogeneous, and the relative contributions of energy restriction, meal timing, and fasting duration remain not completely understood [161]. Overall, TRE may represent a feasible dietary option for selected individuals, but current evidence does not establish a universally optimal eating-window duration or timing. Long-term randomized studies that control or carefully assess energy intake and directly compare early and late eating windows are needed to clarify the independent contribution of meal timing and its interaction with circadian physiology.

Diet composition and meal timing may therefore influence longevity, while socioeconomic and organizational factors, including work schedules, time constraints, poverty, dietary habits, and cultural approaches to fasting, can predispose individuals to disordered eating behaviors such as meal skipping [80].

Overall, the current evidence suggests that caloric restriction may improve body weight, fat mass, insulin sensitivity, and certain markers of systemic inflammation and cardiometabolic health [168]. However, improvements in risk factors or molecular biomarkers should not be taken as proof of a reduced risk of cardiovascular disease or cancer. It is unclear to what extent fasting and CR reduce the long-term risk of these diseases in humans, and this requires further investigation. Similarly, the potential effects of these interventions on neurodegenerative diseases and lifespan extension in humans are not well established. [7].

Notably, the refeeding phase is not simply the return to normal eating; it is a distinct period of metabolic adaptation. Refeeding after prolonged fasting should be gradual, particularly in malnourished or medically fragile patients, because rapid carbohydrate reintroduction can cause hypophosphataemia, hypokalaemia, hypomagnesaemia, thiamine deficiency, and fluid shifts. High-risk patients require electrolyte assessment, thiamine, and clinical monitoring [10.1002/ncp.10474]. In mice, refeeding increases intestinal stem-cell proliferation and can enhance tumour formation in genetically susceptible, Apc-deficient models [169], although this does not demonstrate increased cancer risk in humans [170]. Thus, refeeding should be followed carefully after prolonged fasting, with gradual increases in food intake rather than a sudden large meal [169].

Notably, fasting is contraindicated in individuals with cardiac or renal failure, peptic ulcer disease, uncontrolled diabetes, protein–calorie malnutrition, older adults receiving multiple medications, and individuals at risk of eating disorders. Moreover, excessive caloric restriction (e.g., diets providing 600–800 kcal/day for approximately two months) may lead to a substantial increase in cardiac fat with consequent impairment of myocardial function [171,172].

In conclusion, adopting severe CR strategies, particularly for individuals with chronic diseases or specific nutritional vulnerabilities, requires a careful evaluation of potential benefits and risks in the context of the individual’s clinical situation. Such caution is especially warranted given the growing popularity of these practices outside of clinical and academic settings. Although moderate energy restriction may be a feasible option for some individuals, the available evidence does not establish an optimal degree of caloric restriction for promoting longevity and well-being in humans. Dietary recommendations should therefore be tailored to each individual’s nutritional status, metabolic health, age, treatment requirements, and the potential risks associated with sustained energy restriction.

## Figures and Tables

**Table 1:** Dietary strategies based on fasting and caloric restriction: populations, principal outcomes, levels of evidence, and major limitations.

Dietary Strategy	Population/Experimental Model	Intervention	Principal Outcomes Reported	Study Design/Level of Evidence	Major Limitations
Continuous caloric restriction (CR)	Adults; rodents and other laboratory animal models; non-human primates	Sustained reduction in energy intake, generally without eliminating essential nutrients	In humans, improvements in body weight, adiposity, and selected cardiometabolic outcomes; in animal models, changes in nutrient-sensing pathways, age-related pathology, and lifespan	Human randomized intervention studies and clinical studies; animal experiments, including long-term studies in non-human primates	Human studies provide limited evidence on long-term disease incidence and lifespan. Animal findings vary across species, experimental conditions, and baseline characteristics.
Intermittent fasting (IF)	Adults in clinical and intervention studies; rodent models	Alternation between periods of eating and fasting, using different schedules	Weight reduction and improvements in selected metabolic outcomes; proposed effects on substrate utilization, cellular stress responses, and inflammatory pathways	Human randomized and non-randomized intervention studies; observational studies; animal experiments	Protocols, fasting duration, comparators, and adherence vary considerably. Effects may partly reflect reduced energy intake or weight loss rather than fasting itself.
Alternate-day fasting (ADF)	Adults; rodents	Alternation between fasting or markedly restricted-intake days and days of unrestricted or usual intake	Changes in body weight and metabolic markers; changes in ketogenesis and brain-related pathways have also been reported in preclinical studies	Human intervention studies, including randomized trials; animal experiments	Definitions of fasting days differ. Long-term adherence and comparative effectiveness depend on the population and comparator. Neurobiological findings in rodents cannot be directly extrapolated to humans.
Time-restricted feeding/eating (TRF/TRE)	Adults in clinical and observational studies; animal models	Daily eating window restricted to a defined period, commonly approximately 8–12 h in the protocols discussed	Changes in body weight and selected metabolic outcomes; potential effects of meal timing on circadian regulation and glucose homeostasis	Human randomized and non-randomized intervention studies; observational human studies; animal experiments	Eating-window duration, timing relative to circadian rhythms, diet composition, and total energy intake differ across studies. Observational associations do not establish causality.
Semi-fasting/periodic low-calorie regimens	Adults; preclinical models where applicable	Substantial but incomplete energy restriction on selected days, with intake depending on the protocol	Changes in energy balance and selected metabolic parameters	Human intervention studies and clinical investigations; preclinical evidence for mechanistic outcomes	Protocols are heterogeneous, and results may depend on the severity and duration of restriction, adherence, and the comparator used.
Fasting-mimicking diet (FMD)	Adults in clinical studies; animal models	Periodic, short-term, low-calorie dietary regimen designed to reproduce selected metabolic effects of fasting while providing limited nutrients	Changes in selected metabolic risk markers; proposed effects on nutrient-sensing pathways and regenerative processes	Human intervention studies, including controlled studies where available; animal experiments; and mechanistic investigations	Evidence for long-term clinical outcomes remains limited. Proposed stem-cell and regenerative effects rely substantially on preclinical or mechanistic findings and should not be interpreted as established clinical benefits in humans.
Prolonged fasting/complete fasting	Human physiological and clinical studies; animal models	Abstinence from food for an extended period, with water permitted in complete-fasting protocols	Ketogenesis and shifts in substrate utilization; changes in endocrine and nutrient-sensing pathways. Preclinical studies report effects on autophagy and selected regenerative processes	Human physiological studies and clinical investigations; animal experiments; in vitro or mechanistic studies for specific cellular pathways	Physiological responses depend on fasting duration and individual characteristics. Evidence that molecular adaptations translate into durable clinical benefits is limited; prolonged fasting also requires careful assessment of safety and contraindications.
Protein or amino-acid restriction	Primarily animal models and mechanistic systems; human observational studies for selected associations	Reduction in total protein intake or restriction of specific amino acids	Changes in nutrient-sensing pathways, including GCN2, FGF21, and mTOR-related signaling; effects on metabolism and longevity in some experimental models	Predominantly animal and mechanistic studies; observational human evidence for selected associations	Findings for specific amino acids or experimental diets cannot be generalized to all forms of protein restriction. Human causal evidence for longevity benefits is insufficient to support broad clinical conclusions.

Note: Evidence categories are descriptive and refer to the predominant study designs supporting the findings discussed in this review; they do not represent a formal GRADE assessment. The strength of evidence should be interpreted in relation to the specific outcome, study quality, population, and intervention. Abbreviations: ADF, alternate-day fasting; CR, caloric restriction; FMD, fasting-mimicking diet; IF, intermittent fasting; TRE, time-restricted eating; TRF, time-restricted feeding.

**Table 2:** Evidence for specific mechanistic and long-term outcomes.

Outcome/Research Area	Predominant Evidence in the Literature Discussed	Main Interpretative Limitation
Autophagy and mitochondrial function	Animal models, in vitro/mechanistic studies, and human studies assessing selected physiological or molecular markers	Changes in pathways or biomarkers do not necessarily demonstrate increased autophagic flux, improved tissue function, or clinically meaningful benefits in humans.
Stem-cell function and tissue regeneration	Predominantly animal and mechanistic studies; selected human studies of metabolic responses to CR or fasting-related interventions	Evidence of regenerative effects in experimental models does not establish clinically relevant tissue regeneration or improved outcomes in humans.
Neuroprotection and cognitive function	Predominantly preclinical studies, complemented by human studies assessing selected cognitive or metabolic outcomes	Effects on BDNF, neurogenesis, or neuronal stress responses in animals do not establish prevention or treatment of human neurodegenerative disease.
Cancer-related outcomes	Cell-based and animal studies, together with human observational and clinical research	Mechanistic plausibility and preclinical findings do not establish cancer prevention or therapeutic efficacy in humans. Effects may differ by cancer type, treatment context, and patient characteristics.
Healthspan and lifespan	Experimental evidence from several animal models, long-term non-human primate studies, and limited human evidence	Results in non-human primates are not fully concordant, and improvements in healthspan should be distinguished from lifespan extension. A causal lifespan benefit in humans has not been established.

Note: Evidence categories are descriptive and refer to the predominant study designs supporting the findings discussed in this review; they do not represent a formal GRADE assessment. The strength of evidence should be interpreted in relation to the specific outcome, study quality, population, and intervention. Abbreviations: CR, caloric restriction; BDNF, brain derived neurotrophic factor.

## Data Availability

No new data were created or analyzed in this study. Data sharing is not applicable to this article.

## References

[1] Surugiu R., Iancu M.A., Vintilescu Ș.B., Stepan M.D., Burdusel D., Genunche-Dumitrescu A.V., Dogaru C.-A., Dumitra G.G. (2024). Molecular Mechanisms of Healthy Aging: The Role of Caloric Restriction, Intermittent Fasting, Mediterranean Diet, and Ketogenic Diet—A Scoping Review. Nutrients.

[2] Trepanowski J.F., Canale R.E., Marshall K.E., Kabir M.M., Bloomer R.J. (2011). Impact of Caloric and Dietary Restriction Regimens on Markers of Health and Longevity in Humans and Animals: A Summary of Available Findings. Nutr. J..

[3] Brandhorst S., Levine M.E., Wei M., Shelehchi M., Morgan T.E., Nayak K.S., Dorff T., Hong K., Crimmins E.M., Cohen P. (2024). Fasting-Mimicking Diet Causes Hepatic and Blood Markers Changes Indicating Reduced Biological Age and Disease Risk. Nat. Commun..

[4] Lustig E., Shubrook J.H., Pfotenhauer K.M. (2020). Time-Restricted Feeding and Potential for Type 2 Diabetes Mellitus: A Narrative Review. J. Osteopath. Med..

[5] Trabelsi K., Ammar A., Boujelbane M.A., Puce L., Garbarino S., Scoditti E., Boukhris O., Khanfir S., Clark C.C.T., Glenn J.M. (2022). Religious Fasting and Its Impacts on Individual, Public, and Planetary Health: Fasting as a “Religious Health Asset” for a Healthier, More Equitable, and Sustainable Society. Front. Nutr..

[6] Termannsen A., Varming A., Van Elst C., Bjerre N., Nørgaard O., Hempler N.F., Færch K., Quist J.S. (2023). Feasibility of Time-restricted Eating in Individuals with Overweight, Obesity, Prediabetes, or Type 2 Diabetes: A Systematic Scoping Review. Obesity.

[7] Green C.L., Lamming D.W., Fontana L. (2022). Molecular Mechanisms of Dietary Restriction Promoting Health and Longevity. Nat. Rev. Mol. Cell Biol..

[8] Scanlan S.J., Stoll L.C., Lumm K. (2008). Starving for Change: The Hunger Strike and Nonviolent Action, 1906–2004. Research in Social Movements, Conflicts and Change.

[9] Arden R., Luciano M., Deary I.J., Reynolds C.A., Pedersen N.L., Plassman B.L., McGue M., Christensen K., Visscher P.M. (2016). The Association between Intelligence and Lifespan Is Mostly Genetic. Int. J. Epidemiol..

[10] Mora R.J.F. (1999). Malnutrition: Organic and Functional Consequences. World J. Surg..

[11] Mitchell S.E., Togo J., Green C.L., Derous D., Hambly C., Speakman J.R. (2023). The Effects of Graded Levels of Calorie Restriction: XX. Impact of Long-Term Graded Calorie Restriction on Survival and Body Mass Dynamics in Male C57BL/6J Mice. J. Gerontol. Ser. A.

[12] Shelton H.M.G., Willard J., Oswald J.A. (1986). The Original Natural Hygiene Weight Loss Diet Book.

[13] Luciani L. (1889). Fisiologia Del Digiuno.

[14] Osborne T.B., Mendel L.B., Ferry E.L. (1917). The Effect of Retardation of Growth Upon the Breeding Period and Duration of Life of Rats. Science.

[15] Lindeberg S., Nilsson-Ehle P., Terént A., Vessby B., Scherstén B. (1994). Cardiovascular Risk Factors in a Melanesian Population Apparently Free from Stroke and Ischaemic Heart Disease: The Kitava Study. J. Intern. Med..

[16] Wrench G.T. (2006). The Wheel of Health: The Sources of Long Life and Health Among the Hunza.

[17] Poulain M., Herm A., Errigo A., Chrysohoou C., Legrand R., Passarino G., Stazi M.A., Voutekatis K.G., Gonos E.S., Franceschi C. (2021). Specific Features of the Oldest Old from the Longevity Blue Zones in Ikaria and Sardinia. Mech. Ageing Dev..

[18] Keys A., Brožek J., Henschel A., Mickelsen O., Taylor H.L. (1950). The Biology of Human Starvation. (2 Vols).

[19] Arias Vallejo E. (1956). Dieta de Hambre En La Alimentacion de Los Viejos. Rev. Clínica Española.

[20] Walford R.L., Mock D., Verdery R., MacCallum T. (2002). Calorie Restriction in Biosphere 2: Alterations in Physiologic, Hematologic, Hormonal, and Biochemical Parameters in Humans Restricted for a 2-Year Period. J. Gerontol. Ser. A Biol. Sci. Med. Sci..

[21] Hoddy K.K., Marlatt K.L., Çetinkaya H., Ravussin E. (2020). Intermittent Fasting and Metabolic Health: From Religious Fast to Time-Restricted Feeding. Obesity.

[22] Gillespie L.M., Volaire F.A. (2017). Are Winter and Summer Dormancy Symmetrical Seasonal Adaptive Strategies? The Case of Temperate Herbaceous Perennials. Ann. Bot..

[23] Secor S.M., Carey H.V. (2016). Integrative Physiology of Fasting. Compr. Physiol..

[24] Longo V.D., Mattson M.P. (2014). Fasting: Molecular Mechanisms and Clinical Applications. Cell Metab..

[25] Bramble D.M., Lieberman D.E. (2004). Endurance Running and the Evolution of Homo. Nature.

[26] Mattson M.P. (2015). Lifelong Brain Health Is a Lifelong Challenge: From Evolutionary Principles to Empirical Evidence. Ageing Res. Rev..

[27] Mattson M.P., Moehl K., Ghena N., Schmaedick M., Cheng A. (2018). Intermittent Metabolic Switching, Neuroplasticity and Brain Health. Nat. Rev. Neurosci..

[28] Mattson M.P., Arumugam T.V. (2018). Hallmarks of Brain Aging: Adaptive and Pathological Modification by Metabolic States. Cell Metab..

[29] Barañano K.W., Hartman A.L. (2008). The Ketogenic Diet: Uses in Epilepsy and Other Neurologic Illnesses. Curr. Treat. Options Neurol..

[30] Van Der Auwera I., Wera S., Van Leuven F., Henderson S.T. (2005). A Ketogenic Diet Reduces Amyloid Beta 40 and 42 in a Mouse Model of Alzheimer’s Disease. Nutr. Metab..

[31] Lee J., Seroogy K.B., Mattson M.P. (2002). Dietary Restriction Enhances Neurotrophin Expression and Neurogenesis in the Hippocampus of Adult Mice. J. Neurochem..

[32] Cahill G.F. (2006). Fuel Metabolism in Starvation. Annu. Rev. Nutr..

[33] García-Rodríguez D., Giménez-Cassina A. (2021). Ketone Bodies in the Brain Beyond Fuel Metabolism: From Excitability to Gene Expression and Cell Signaling. Front. Mol. Neurosci..

[34] Zauner C., Schneeweiss B., Kranz A., Madl C., Ratheiser K., Kramer L., Roth E., Schneider B., Lenz K. (2000). Resting Energy Expenditure in Short-Term Starvation Is Increased as a Result of an Increase in Serum Norepinephrine. Am. J. Clin. Nutr..

[35] Prentice A.M. (2005). Starvation in Humans: Evolutionary Background and Contemporary Implications. Mech. Ageing Dev..

[36] Hursting S.D., Dunlap S.M., Ford N.A., Hursting M.J., Lashinger L.M. (2013). Calorie Restriction and Cancer Prevention: A Mechanistic Perspective. Cancer Metab..

[37] Roth G.S., Lane M.A., Ingram D.K., Mattison J.A., Elahi D., Tobin J.D., Muller D., Metter E.J. (2002). Biomarkers of Caloric Restriction May Predict Longevity in Humans. Science.

[38] McGolphin Shelton H. (1953). The Hygienic System: Orthobionomics.

[39] Solianik R., Sujeta A., Terentjevienė A., Skurvydas A. (2016). Effect of 48 h Fasting on Autonomic Function, Brain Activity, Cognition, and Mood in Amateur Weight Lifters. BioMed Res. Int..

[40] Wang Y., Wu R. (2022). The Effect of Fasting on Human Metabolism and Psychological Health. Dis. Markers.

[41] Watkins E., Serpell L. (2016). The Psychological Effects of Short-Term Fasting in Healthy Women. Front. Nutr..

[42] Bamberg C., Moreau D. (2025). Acute Effects of Fasting on Cognitive Performance: A Systematic Review and Meta-Analysis. Psychol. Bull..

[43] Tognozzi A., Carli F., Abdelkarim S., Cornuti S., Damiani F., Giuliano M.G., Miniati A., Nasisi M., De Benedictis L., Ashtiani K.C. (2026). Succinate Modulation as a Biochemical Correlate of Metabolic and Neurobehavioral Changes Associated With Intermittent Fasting in Obesity. Acta Physiol..

[44] Martucci M., Ostan R., Biondi F., Bellavista E., Fabbri C., Bertarelli C., Salvioli S., Capri M., Franceschi C., Santoro A. (2017). Mediterranean Diet and Inflammaging within the Hormesis Paradigm. Nutr. Rev..

[45] Gao L., Wang J., Sekhar K.R., Yin H., Yared N.F., Schneider S.N., Sasi S., Dalton T.P., Anderson M.E., Chan J.Y. (2007). Novel N-3 Fatty Acid Oxidation Products Activate Nrf2 by Destabilizing the Association between Keap1 and Cullin3. J. Biol. Chem..

[46] Storoschuk K.L., Lesiuk D., Nuttall J., LeBouedec M., Khansari A., Islam H., Gurd B.J. (2024). Impact of Fasting on the AMPK and PGC-1α Axis in Rodent and Human Skeletal Muscle: A Systematic Review. Metabolism.

[47] Ventura-Clapier R., Garnier A., Veksler V. (2008). Transcriptional Control of Mitochondrial Biogenesis: The Central Role of PGC-1. Cardiovasc. Res..

[48] Anson R.M., Guo Z., De Cabo R., Iyun T., Rios M., Hagepanos A., Ingram D.K., Lane M.A., Mattson M.P. (2003). Intermittent Fasting Dissociates Beneficial Effects of Dietary Restriction on Glucose Metabolism and Neuronal Resistance to Injury from Calorie Intake. Proc. Natl. Acad. Sci. USA.

[49] Mei G., Pan H., Zheng J., Chen K., Zheng W., Xu H., Liu Z., Lin W., Lin Z., Zhang M. (2026). Crosstalk between Circadian Rhythm and Neuroinflammation. Neuroendocrinology.

[50] Reytor-González C., Simancas-Racines D., Román-Galeano N.M., Annunziata G., Galasso M., Zambrano-Villacres R., Verde L., Muscogiuri G., Frias-Toral E., Barrea L. (2025). Chrononutrition and Energy Balance: How Meal Timing and Circadian Rhythms Shape Weight Regulation and Metabolic Health. Nutrients.

[51] Vollmers C., Gill S., DiTacchio L., Pulivarthy S.R., Le H.D., Panda S. (2009). Time of Feeding and the Intrinsic Circadian Clock Drive Rhythms in Hepatic Gene Expression. Proc. Natl. Acad. Sci. USA.

[52] Hirayama M., Mure L.S., Panda S. (2018). Circadian Regulation of Energy Intake in Mammals. Curr. Opin. Physiol..

[53] Pardi D., Buman M., Black J., Lammers G.J., Zeitzer J.M. (2017). Eating Decisions Based on Alertness Levels After a Single Night of Sleep Manipulation: A Randomized Clinical Trial. Sleep.

[54] Thun E., Bjorvatn B., Flo E., Harris A., Pallesen S. (2015). Sleep, Circadian Rhythms, and Athletic Performance. Sleep Med. Rev..

[55] Chaix A., Zarrinpar A., Miu P., Panda S. (2014). Time-Restricted Feeding Is a Preventative and Therapeutic Intervention against Diverse Nutritional Challenges. Cell Metab..

[56] Filipski E., King V.M., Li X., Granda T.G., Mormont M.-C., Liu X., Claustrat B., Hastings M.H., Lévi F. (2002). Host Circadian Clock as a Control Point in Tumor Progression. J. Natl. Cancer Inst..

[57] Rudic R.D., McNamara P., Curtis A.-M., Boston R.C., Panda S., Hogenesch J.B., FitzGerald G.A. (2004). BMAL1 and CLOCK, Two Essential Components of the Circadian Clock, Are Involved in Glucose Homeostasis. PLoS Biol..

[58] Arble D.M., Bass J., Laposky A.D., Vitaterna M.H., Turek F.W. (2009). Circadian Timing of Food Intake Contributes to Weight Gain. Obesity.

[59] Turnbaugh P.J. (2017). Microbes and Diet-Induced Obesity: Fast, Cheap, and Out of Control. Cell Host Microbe.

[60] Hu X., Xia K., Dai M., Han X., Yuan P., Liu J., Liu S., Jia F., Chen J., Jiang F. (2023). Intermittent Fasting Modulates the Intestinal Microbiota and Improves Obesity and Host Energy Metabolism. npj Biofilms Microbiomes.

[61] Khonsary S. (2017). Guyton and Hall: Textbook of Medical Physiology. Surg. Neurol. Int..

[62] Vasim I., Majeed C.N., DeBoer M.D. (2022). Intermittent Fasting and Metabolic Health. Nutrients.

[63] Veech R.L., Chance B., Kashiwaya Y., Lardy H.A., Cahill G.F. (2001). Ketone Bodies, Potential Therapeutic Uses. IUBMB Life.

[64] Haymond M.W., Karl I.E., Clarke W.L., Pagliara A.S., Santiago J.V. (1982). Differences in Circulating Gluconeogenic Substrates during Short-Term Fasting in Men, Women, and Children. Metabolism.

[65] Schübel R., Nattenmüller J., Sookthai D., Nonnenmacher T., Graf M.E., Riedl L., Schlett C.L., von Stackelberg O., Johnson T., Nabers D. (2018). Effects of Intermittent and Continuous Calorie Restriction on Body Weight and Metabolism over 50 Wk: A Randomized Controlled Trial. Am. J. Clin. Nutr..

[66] Meas T., Taboulet P., Sobngwi E., Gautier J. (2005). Is Capillary Ketone Determination Useful in Clinical Practice? In Which Circumstances?. Diabetes Metab..

[67] Stewart W.K., Fleming L.W. (1973). Features of a Successful Therapeutic Fast of 382 Days’ Duration. Postgrad. Med. J..

[68] Sailasuta N., Harris K.C., Tran T.T., Abulseoud O., Ross B.D. (2014). Impact of Fasting on Human Brain Acid-base Homeostasis Using Natural Abundance ^13^C and ^31^P MRS. Magn. Reson. Imaging.

[69] Lloyd-Mostyn R.H., Lord P.S., Glover R., West C., Gilliland I.C. (1970). Uric Acid Metabolism in Starvation. Ann. Rheum. Dis..

[70] Murphy R., Shipman K.H. (1963). HYPERURICEMIA DURING TOTAL FASTING: RENAL FACTORS. Arch. Intern. Med..

[71] Sautin Y.Y., Johnson R.J. (2008). Uric Acid: The Oxidant-Antioxidant Paradox. Nucleosides Nucleotides Nucleic Acids.

[72] Doblado M., Moley K.H. (2009). Facilitative Glucose Transporter 9, a Unique Hexose and Urate Transporter. Am. J. Physiol. Endocrinol. Metab..

[73] Harvie M.N., Pegington M., Mattson M.P., Frystyk J., Dillon B., Evans G., Cuzick J., Jebb S.A., Martin B., Cutler R.G. (2011). The Effects of Intermittent or Continuous Energy Restriction on Weight Loss and Metabolic Disease Risk Markers: A Randomized Trial in Young Overweight Women. Int. J. Obes..

[74] Moro T., Tinsley G., Bianco A., Marcolin G., Pacelli Q.F., Battaglia G., Palma A., Gentil P., Neri M., Paoli A. (2016). Effects of Eight Weeks of Time-Restricted Feeding (16/8) on Basal Metabolism, Maximal Strength, Body Composition, Inflammation, and Cardiovascular Risk Factors in Resistance-Trained Males. J. Transl. Med..

[75] Ravussin E., Redman L.M., Rochon J., Das S.K., Fontana L., Kraus W.E., Romashkan S., Williamson D.A., Meydani S.N., Villareal D.T. (2015). A 2-Year Randomized Controlled Trial of Human Caloric Restriction: Feasibility and Effects on Predictors of Health Span and Longevity. J. Gerontol. Ser. A Biomed. Sci. Med. Sci..

[76] Farooqi I.S., Keogh J.M., Kamath S., Jones S., Gibson W.T., Trussell R., Jebb S.A., Lip G.Y.H., O’Rahilly S. (2001). Partial Leptin Deficiency and Human Adiposity. Nature.

[77] Merl V., Peters A., Oltmanns K.M., Kern W., Born J., Fehm H.L., Schultes B. (2005). Serum Adiponectin Concentrations during a 72-Hour Fast in over- and Normal-Weight Humans. Int. J. Obes..

[78] Ho K.Y., Veldhuis J.D., Johnson M.L., Furlanetto R., Evans W.S., Alberti K.G., Thorner M.O. (1988). Fasting Enhances Growth Hormone Secretion and Amplifies the Complex Rhythms of Growth Hormone Secretion in Man. J. Clin. Investig..

[79] Fontana L., Partridge L. (2015). Promoting Health and Longevity through Diet: From Model Organisms to Humans. Cell.

[80] Gubbi S., Quipildor G.F., Barzilai N., Huffman D.M., Milman S. (2018). 40 YEARS of IGF1: IGF1: The Jekyll and Hyde of the Aging Brain. J. Mol. Endocrinol..

[81] Heath H. (2017). The Telomere Effect: A Revolutionary Approach to Living Younger, Healthier, Longer. Nurs. Stand..

[82] Wang C., Maddick M., Miwa S., Jurk D., Czapiewski R., Saretzki G., Langie S.A.S., Godschalk R.W.L., Cameron K., Von Zglinicki T. (2010). Adult-Onset, Short-Term Dietary Restriction Reduces Cell Senescence in Mice. Aging.

[83] Sagheddu C., Melis M. (2015). Individual Differences and Vulnerability to Drug Addiction: A Focus on the Endocannabinoid System. CNS Neurol. Disord.-Drug Targets.

[84] Melis M., Carta G., Pistis M., Banni S. (2013). Physiological Role of Peroxisome Proliferator-Activated Receptors Type Alpha on Dopamine Systems. CNS Neurol. Disord.-Drug Targets.

[85] Mauvais-Jarvis F., Clegg D.J., Hevener A.L. (2013). The Role of Estrogens in Control of Energy Balance and Glucose Homeostasis. Endocr. Rev..

[86] Mauvais-Jarvis F. (2024). Sex Differences in Energy Metabolism: Natural Selection, Mechanisms and Consequences. Nat. Rev. Nephrol..

[87] Waly T.A., Nugroho B.S. (2025). The Analysis Study Effect of Intermittent Fasting on Female Reproductive Hormones and Menstrual Cycle: A Comprehensive Systematic Review. Int. J. Med. Sci. Health.

[88] Lin S., Cienfuegos S., Ezpeleta M., Pavlou V., Runchey M.-C., Varady K.A. (2024). Effect of Time Restricted Eating versus Daily Calorie Restriction on Sex Hormones in Males and Females with Obesity. Eur. J. Clin. Nutr..

[89] Cienfuegos S., Corapi S., Gabel K., Ezpeleta M., Kalam F., Lin S., Pavlou V., Varady K.A. (2022). Effect of Intermittent Fasting on Reproductive Hormone Levels in Females and Males: A Review of Human Trials. Nutrients.

[90] Bar-Peled L., Sabatini D.M. (2014). Regulation of mTORC1 by Amino Acids. Trends Cell Biol..

[91] Liu G.Y., Sabatini D.M. (2020). mTOR at the Nexus of Nutrition, Growth, Ageing and Disease. Nat. Rev. Mol. Cell Biol..

[92] Mercken E.M., Crosby S.D., Lamming D.W., JeBailey L., Krzysik-Walker S., Villareal D.T., Capri M., Franceschi C., Zhang Y., Becker K. (2013). Calorie Restriction in Humans Inhibits the PI3K/AKT Pathway and Induces a Younger Transcription Profile. Aging Cell.

[93] Srivastava A., Lu J., Gadalla D.S., Hendrich O., Grönke S., Partridge L. (2022). The Role of GCN2 Kinase in Mediating the Effects of Amino Acids on Longevity and Feeding Behaviour in Drosophila. Front. Aging.

[94] Bunpo P., Cundiff J.K., Reinert R.B., Wek R.C., Aldrich C.J., Anthony T.G. (2010). The eIF2 Kinase GCN2 Is Essential for the Murine Immune System to Adapt to Amino Acid Deprivation by Asparaginase. J. Nutr..

[95] Ye J., Kumanova M., Hart L.S., Sloane K., Zhang H., De Panis D.N., Bobrovnikova-Marjon E., Diehl J.A., Ron D., Koumenis C. (2010). The GCN2-ATF4 Pathway Is Critical for Tumour Cell Survival and Proliferation in Response to Nutrient Deprivation. EMBO J..

[96] Ravindran R., Loebbermann J., Nakaya H.I., Khan N., Ma H., Gama L., Machiah D.K., Lawson B., Hakimpour P., Wang Y. (2016). The Amino Acid Sensor GCN2 Controls Gut Inflammation by Inhibiting Inflammasome Activation. Nature.

[97] Laeger T., Henagan T.M., Albarado D.C., Redman L.M., Bray G.A., Noland R.C., Münzberg H., Hutson S.M., Gettys T.W., Schwartz M.W. (2014). FGF21 Is an Endocrine Signal of Protein Restriction. J. Clin. Investig..

[98] Kharitonenkov A., Shiyanova T.L., Koester A., Ford A.M., Micanovic R., Galbreath E.J., Sandusky G.E., Hammond L.J., Moyers J.S., Owens R.A. (2005). FGF-21 as a Novel Metabolic Regulator. J. Clin. Investig..

[99] von Holstein-Rathlou S., BonDurant L.D., Peltekian L., Naber M.C., Yin T.C., Claflin K.E., Urizar A.I., Madsen A.N., Ratner C., Holst B. (2016). FGF21 Mediates Endocrine Control of Simple Sugar Intake and Sweet Taste Preference by the Liver. Cell Metab..

[100] Eldridge M.J.G., Pereira J.M., Impens F., Hamon M.A. (2020). Active Nuclear Import of the Deacetylase Sirtuin-2 Is Controlled by Its C-Terminus and Importins. Sci. Rep..

[101] Satoh A., Brace C.S., Ben-Josef G., West T., Wozniak D.F., Holtzman D.M., Herzog E.D., Imai S. (2010). SIRT1 Promotes the Central Adaptive Response to Diet Restriction through Activation of the Dorsomedial and Lateral Nuclei of the Hypothalamus. J. Neurosci..

[102] Marques F.Z., Markus M.A., Morris B.J. (2010). Hormesis as a Pro-Healthy Aging Intervention in Human Beings?. Dose-Response.

[103] Civitarese A.E., Carling S., Heilbronn L.K., Hulver M.H., Ukropcova B., Deutsch W.A., Smith S.R., Ravussin E. (2007). Calorie Restriction Increases Muscle Mitochondrial Biogenesis in Healthy Humans. PLoS Med..

[104] Lutz M.I., Milenkovic I., Regelsberger G., Kovacs G.G. (2014). Distinct Patterns of Sirtuin Expression During Progression of Alzheimer’s Disease. Neuromol. Med..

[105] Pedersen S.B., Ølholm J., Paulsen S.K., Bennetzen M.F., Richelsen B. (2008). Low Sirt1 Expression, Which Is Upregulated by Fasting, in Human Adipose Tissue from Obese Women. Int. J. Obes..

[106] Anderson R.M., Barger J.L., Edwards M.G., Braun K.H., O’Connor C.E., Prolla T.A., Weindruch R. (2008). Dynamic Regulation of PGC-1α Localization and Turnover Implicates Mitochondrial Adaptation in Calorie Restriction and the Stress Response. Aging Cell.

[107] Hetz C., Zhang K., Kaufman R.J. (2020). Mechanisms, Regulation and Functions of the Unfolded Protein Response. Nat. Rev. Mol. Cell Biol..

[108] Nisoli E., Tonello C., Cardile A., Cozzi V., Bracale R., Tedesco L., Falcone S., Valerio A., Cantoni O., Clementi E. (2005). Calorie Restriction Promotes Mitochondrial Biogenesis by Inducing the Expression of eNOS. Science.

[109] Il’yasova D., Fontana L., Bhapkar M., Pieper C.F., Spasojevic I., Redman L.M., Das S.K., Huffman K.M., Kraus W.E. (2018). The CALERIE Study Investigators Effects of 2 Years of Caloric Restriction on Oxidative Status Assessed by Urinary F2-isoprostanes: The CALERIE 2 Randomized Clinical Trial. Aging Cell.

[110] Meyer T.E., Kovács S.J., Ehsani A.A., Klein S., Holloszy J.O., Fontana L. (2006). Long-Term Caloric Restriction Ameliorates the Decline in Diastolic Function in Humans. J. Am. Coll. Cardiol..

[111] Yang L., Licastro D., Cava E., Veronese N., Spelta F., Rizza W., Bertozzi B., Villareal D.T., Hotamisligil G.S., Holloszy J.O. (2016). Long-Term Calorie Restriction Enhances Cellular Quality-Control Processes in Human Skeletal Muscle. Cell Rep..

[112] El Kebbaj Z., Andreoletti P., Mountassif D., Kabine M., Schohn H., Dauça M., Latruffe N., El Kebbaj M.S., Cherkaoui-Malki M. (2009). Differential Regulation of Peroxisome Proliferator-Activated Receptor (PPAR)-A1 and Truncated PPARα2 as an Adaptive Response to Fasting in the Control of Hepatic Peroxisomal Fatty Acid β-Oxidation in the Hibernating Mammal. Endocrinology.

[113] Mana M.D., Kuo E.Y.-S., Yilmaz Ö.H. (2017). Dietary Regulation of Adult Stem Cells. Curr. Stem Cell Rep..

[114] Kenyon C.J. (2010). The Genetics of Ageing. Nature.

[115] Hanjani N., Vafa M. (2018). Protein Restriction, Epigenetic Diet, Intermittent Fasting as New Approaches for Preventing Age-Associated Diseases. Int. J. Prev. Med..

[116] Jin K., Yao Z., Van Velthoven C.T.J., Kaplan E.S., Glattfelder K., Barlow S.T., Boyer G., Carey D., Casper T., Chakka A.B. (2025). Brain-Wide Cell-Type-Specific Transcriptomic Signatures of Healthy Ageing in Mice. Nature.

[117] López-Otín C., Blasco M.A., Partridge L., Serrano M., Kroemer G. (2013). The Hallmarks of Aging. Cell.

[118] Lamming D.W. (2014). Diminished mTOR Signaling: A Common Mode of Action for Endocrine Longevity Factors. SpringerPlus.

[119] Cheng C.-W., Adams G.B., Perin L., Wei M., Zhou X., Lam B.S., Da Sacco S., Mirisola M., Quinn D.I., Dorff T.B. (2014). Prolonged Fasting Reduces IGF-1/PKA to Promote Hematopoietic-Stem-Cell-Based Regeneration and Reverse Immunosuppression. Cell Stem Cell.

[120] Mattison J.A., Colman R.J., Beasley T.M., Allison D.B., Kemnitz J.W., Roth G.S., Ingram D.K., Weindruch R., De Cabo R., Anderson R.M. (2017). Caloric Restriction Improves Health and Survival of Rhesus Monkeys. Nat. Commun..

[121] Colman R.J., Anderson R.M., Johnson S.C., Kastman E.K., Kosmatka K.J., Beasley T.M., Allison D.B., Cruzen C., Simmons H.A., Kemnitz J.W. (2009). Caloric Restriction Delays Disease Onset and Mortality in Rhesus Monkeys. Science.

[122] Maegawa S., Lu Y., Tahara T., Lee J.T., Madzo J., Liang S., Jelinek J., Colman R.J., Issa J.-P.J. (2017). Caloric Restriction Delays Age-Related Methylation Drift. Nat. Commun..

[123] Mihaylova M.M., Cheng C.-W., Cao A.Q., Tripathi S., Mana M.D., Bauer-Rowe K.E., Abu-Remaileh M., Clavain L., Erdemir A., Lewis C.A. (2018). Fasting Activates Fatty Acid Oxidation to Enhance Intestinal Stem Cell Function during Homeostasis and Aging. Cell Stem Cell.

[124] Richardson N.E., Konon E.N., Schuster H.S., Mitchell A.T., Boyle C., Rodgers A.C., Finke M., Haider L.R., Yu D., Flores V. (2021). Lifelong Restriction of Dietary Branched-Chain Amino Acids Has Sex-Specific Benefits for Frailty and Life Span in Mice. Nat. Aging.

[125] Mittendorfer B., Klein S., Fontana L. (2020). A Word of Caution against Excessive Protein Intake. Nat. Rev. Endocrinol..

[126] Smith Z.D., Sindhu C., Meissner A. (2016). Molecular Features of Cellular Reprogramming and Development. Nat. Rev. Mol. Cell Biol..

[127] Willcox B.J., Willcox D.C., Todoriki H., Fujiyoshi A., Yano K., He Q., Curb J.D., Suzuki M. (2007). Caloric Restriction, the Traditional Okinawan Diet, and Healthy Aging: The Diet of the World’s Longest-Lived People and Its Potential Impact on Morbidity and Life Span. Ann. N. Y. Acad. Sci..

[128] Rondanelli M., Cereda E., Klersy C., Faliva M.A., Peroni G., Nichetti M., Gasparri C., Iannello G., Spadaccini D., Infantino V. (2020). Improving Rehabilitation in Sarcopenia: A Randomized-controlled Trial Utilizing a Muscle-targeted Food for Special Medical Purposes. J. Cachexia Sarcopenia Muscle.

[129] Miller R.A., Buehner G., Chang Y., Harper J.M., Sigler R., Smith-Wheelock M. (2005). Methionine-deficient Diet Extends Mouse Lifespan, Slows Immune and Lens Aging, Alters Glucose, T4, IGF-I and Insulin Levels, and Increases Hepatocyte MIF Levels and Stress Resistance. Aging Cell.

[130] Haws S.A., Yu D., Ye C., Wille C.K., Nguyen L.C., Krautkramer K.A., Tomasiewicz J.L., Yang S.E., Miller B.R., Liu W.H. (2020). Methyl-Metabolite Depletion Elicits Adaptive Responses to Support Heterochromatin Stability and Epigenetic Persistence. Mol. Cell.

[131] Douris N., Stevanovic D.M., Fisher F.M., Cisu T.I., Chee M.J., Nguyen N.L., Zarebidaki E., Adams A.C., Kharitonenkov A., Flier J.S. (2015). Central Fibroblast Growth Factor 21 Browns White Fat via Sympathetic Action in Male Mice. Endocrinology.

[132] Ward N.P., Yoon S.J., Flynn T., Sherwood A.M., Olley M.A., Madej J., DeNicola G.M. (2024). Mitochondrial Respiratory Function Is Preserved under Cysteine Starvation via Glutathione Catabolism in NSCLC. Nat. Commun..

[133] Ommati M.M., Amjadinia A., Mousavi K., Azarpira N., Jamshidzadeh A., Heidari R. (2021). N-Acetyl Cysteine Treatment Mitigates Biomarkers of Oxidative Stress in Different Tissues of Bile Duct Ligated Rats. Stress.

[134] Cummings N.E., Williams E.M., Kasza I., Konon E.N., Schaid M.D., Schmidt B.A., Poudel C., Sherman D.S., Yu D., Arriola Apelo S.I. (2018). Restoration of Metabolic Health by Decreased Consumption of Branched-chain Amino Acids. J. Physiol..

[135] Solon-Biet S.M., Cogger V.C., Pulpitel T., Wahl D., Clark X., Bagley E.E., Gregoriou G.C., Senior A.M., Wang Q.-P., Brandon A.E. (2019). Branched-Chain Amino Acids Impact Health and Lifespan Indirectly via Amino Acid Balance and Appetite Control. Nat. Metab..

[136] Fontana L., Cummings N.E., Arriola Apelo S.I., Neuman J.C., Kasza I., Schmidt B.A., Cava E., Spelta F., Tosti V., Syed F.A. (2016). Decreased Consumption of Branched-Chain Amino Acids Improves Metabolic Health. Cell Rep..

[137] Imai S., Armstrong C.M., Kaeberlein M., Guarente L. (2000). Transcriptional Silencing and Longevity Protein Sir2 Is an NAD-Dependent Histone Deacetylase. Nature.

[138] Ramos-Chávez L.A., Roldán-Roldán G., García-Juárez B., González-Esquivel D., Pérez De La Cruz G., Pineda B., Ramírez-Ortega D., García Muñoz I., Jiménez Herrera B., Ríos C. (2018). Low Serum Tryptophan Levels as an Indicator of Global Cognitive Performance in Nondemented Women over 50 Years of Age. Oxidative Med. Cell. Longev..

[139] Yap Y.W., Rusu P.M., Chan A.Y., Fam B.C., Jungmann A., Solon-Biet S.M., Barlow C.K., Creek D.J., Huang C., Schittenhelm R.B. (2020). Restriction of Essential Amino Acids Dictates the Systemic Metabolic Response to Dietary Protein Dilution. Nat. Commun..

[140] Qu Q., Chen Y., Wang Y., Long S., Wang W., Yang H.-Y., Li M., Tian X., Wei X., Liu Y.-H. (2025). Lithocholic Acid Phenocopies Anti-Ageing Effects of Calorie Restriction. Nature.

[141] Sato Y., Atarashi K., Plichta D.R., Arai Y., Sasajima S., Kearney S.M., Suda W., Takeshita K., Sasaki T., Okamoto S. (2021). Novel Bile Acid Biosynthetic Pathways Are Enriched in the Microbiome of Centenarians. Nature.

[142] Johnson J.B., Summer W., Cutler R.G., Martin B., Hyun D.-H., Dixit V.D., Pearson M., Nassar M., Tellejohan R., Maudsley S. (2007). Alternate Day Calorie Restriction Improves Clinical Findings and Reduces Markers of Oxidative Stress and Inflammation in Overweight Adults with Moderate Asthma. Free Radic. Biol. Med..

[143] Dong T.A., Sandesara P.B., Dhindsa D.S., Mehta A., Arneson L.C., Dollar A.L., Taub P.R., Sperling L.S. (2020). Intermittent Fasting: A Heart Healthy Dietary Pattern?. Am. J. Med..

[144] Varady K.A., Cienfuegos S., Ezpeleta M., Gabel K. (2021). Cardiometabolic Benefits of Intermittent Fasting. Annu. Rev. Nutr..

[145] Antal B.B., Van Nieuwenhuizen H., Chesebro A.G., Strey H.H., Jones D.T., Clarke K., Weistuch C., Ratai E.-M., Dill K.A., Mujica-Parodi L.R. (2025). Brain Aging Shows Nonlinear Transitions, Suggesting a Midlife “Critical Window” for Metabolic Intervention. Proc. Natl. Acad. Sci. USA.

[146] Guo L., Xi Y., Jin W., Yuan H., Qin G., Chen S., Zhang L., Liu Y., Cheng X., Liu W. (2024). A 5:2 Intermittent Fasting Meal Replacement Diet and Glycemic Control for Adults With Diabetes: The EARLY Randomized Clinical Trial. JAMA Netw. Open.

[147] Obermayer A., Tripolt N.J., Pferschy P.N., Kojzar H., Aziz F., Müller A., Schauer M., Oulhaj A., Aberer F., Sourij C. (2023). Efficacy and Safety of Intermittent Fasting in People With Insulin-Treated Type 2 Diabetes (INTERFAST-2)—A Randomized Controlled Trial. Diabetes Care.

[148] Malik S., Bhanji A., Abuleiss H., Hamer R., Shah S.H., Rashad R., Junglee N., Waqar S., Ghouri N. (2021). Effects of Fasting on Patients with Chronic Kidney Disease during Ramadan and Practical Guidance for Healthcare Professionals. Clin. Kidney J..

[149] Ahmad S., Chowdhury T.A. (2019). Fasting during Ramadan in People with Chronic Kidney Disease: A Review of the Literature. Ther. Adv. Endocrinol..

[150] Murphy C.H., Hector A.J., Phillips S.M. (2015). Considerations for Protein Intake in Managing Weight Loss in Athletes. Eur. J. Sport Sci..

[151] Zhang H., Li M., Mo L., Luo J., Shen Q., Quan W. (2023). Association between Western Dietary Patterns, Typical Food Groups, and Behavioral Health Disorders: An Updated Systematic Review and Meta-Analysis of Observational Studies. Nutrients.

[152] Mountjoy M., Ackerman K.E., Bailey D.M., Burke L.M., Constantini N., Hackney A.C., Heikura I.A., Melin A., Pensgaard A.M., Stellingwerff T. (2023). 2023 International Olympic Committee’s (IOC) Consensus Statement on Relative Energy Deficiency in Sport (REDs). Br. J. Sports Med..

[153] Hay P. (2020). Current Approach to Eating Disorders: A Clinical Update. Intern. Med. J..

[154] Colombarolli M.S., De Oliveira J., Cordás T.A. (2022). Craving for Carbs: Food Craving and Disordered Eating in Low-Carb Dieters and Its Association with Intermittent Fasting. Eat. Weight Disord..

[155] American Psychiatric Association (2023). Guideline Statement Summary. The American Psychiatric Association Practice Guideline for the Treatment of Patients with Eating Disorders.

[156] Shabkhizan R., Haiaty S., Moslehian M.S., Bazmani A., Sadeghsoltani F., Saghaei Bagheri H., Rahbarghazi R., Sakhinia E. (2023). The Beneficial and Adverse Effects of Autophagic Response to Caloric Restriction and Fasting. Adv. Nutr..

[157] Rahimah S., Tallei T.E., Savitri M., Yamada C., Kim H.J., Choi M., Park M.N., Ophinni Y., Kim B. (2026). Molecular Basis of Precision Nutrition: Food Components, Microbiome-Derived Metabolites, and Multi-Omics Modeling. Food Chem. Mol. Sci..

[158] Cabib S., Orsini C., Moal M.L., Piazza P.V. (2000). Abolition and Reversal of Strain Differences in Behavioral Responses to Drugs of Abuse After a Brief Experience. Science.

[159] Satija A., Yu E., Willett W.C., Hu F.B. (2015). Understanding Nutritional Epidemiology and Its Role in Policy. Adv. Nutr..

[160] St-Onge M.-P., Ard J., Baskin M.L., Chiuve S.E., Johnson H.M., Kris-Etherton P., Varady K. (2017). Meal Timing and Frequency: Implications for Cardiovascular Disease Prevention: A Scientific Statement From the American Heart Association. Circulation.

[161] Quist J.S., Pedersen H.E., Jensen M.M., Clemmensen K.K.B., Bjerre N., Ekblond T.S., Uldal S., Størling J., Wewer Albrechtsen N.J., Holst J.J. (2024). Effects of 3 Months of 10-h per-Day Time-Restricted Eating and 3 Months of Follow-up on Bodyweight and Cardiometabolic Health in Danish Individuals at High Risk of Type 2 Diabetes: The RESET Single-Centre, Parallel, Superiority, Open-Label, Randomised Controlled Trial. Lancet Healthy Longev..

[162] Lotti S., Gallosti S., De Amicis R., Bertoli S., Colombini B., Mazzoccoli G., Dinu M. (2025). Controversies and Perspectives of Time-Qualified Dietary Interventions. Nutrients.

[163] Raji O.E., Kyeremah E.B., Sears D.D., St-Onge M.-P., Makarem N. (2024). Chrononutrition and Cardiometabolic Health: An Overview of Epidemiological Evidence and Key Future Research Directions. Nutrients.

[164] Lee M.B., Hill C.M., Bitto A., Kaeberlein M. (2021). Antiaging Diets: Separating Fact from Fiction. Science.

[165] Manoogian E.N.C., Chaix A., Panda S. (2019). When to Eat: The Importance of Eating Patterns in Health and Disease. J. Biol. Rhythm..

[166] Sun Y., Rong S., Liu B., Du Y., Wu Y., Chen L., Xiao Q., Snetselaar L., Wallace R., Bao W. (2023). Meal Skipping and Shorter Meal Intervals Are Associated with Increased Risk of All-Cause and Cardiovascular Disease Mortality among US Adults. J. Acad. Nutr. Diet..

[167] Mao Z., Grant H., Kritchevsky S.B., Newman A.B., Farsijani S. (2025). Association of Eating Window With Mortality Among US Adults: Insights From a Nationally Representative Study. Aging Cell.

[168] Matta L., Weber P., Erener S., Walth-Hummel A., Hass D., Bühler L.K., Klepac K., Szendroedi J., Guerra J., Rohm M. (2025). Chronic Intermittent Fasting Impairs β Cell Maturation and Function in Adolescent Mice. Cell Rep..

[169] Imada S., Khawaled S., Shin H., Meckelmann S.W., Whittaker C.A., Corrêa R.O., Alquati C., Lu Y., Tie G., Pradhan D. (2024). Short-Term Post-Fast Refeeding Enhances Intestinal Stemness via Polyamines. Nature.

[170] Imada S., Shin H., Khawaled S., Meckelmann S., Whittaker C., Correa R., Pradhan D., Calibasi G., Nascentes L.N., Allies G. (2023). Post-Fast Refeeding Enhances Intestinal Stem Cell-Mediated Regeneration and Tumourigenesis through mTORC1-Dependent Polyamine Synthesis. Res. Sq..

[171] Van Der Meer R.W., Hammer S., Smit J.W.A., Frölich M., Bax J.J., Diamant M., Rijzewijk L.J., De Roos A., Romijn J.A., Lamb H.J. (2007). Short-Term Caloric Restriction Induces Accumulation of Myocardial Triglycerides and Decreases Left Ventricular Diastolic Function in Healthy Subjects. Diabetes.

[172] Rayner J.J., Abdesselam I., Peterzan M.A., Akoumianakis I., Akawi N., Antoniades C., Tomlinson J.W., Neubauer S., Rider O.J. (2019). Very Low Calorie Diets Are Associated with Transient Ventricular Impairment before Reversal of Diastolic Dysfunction in Obesity. Int. J. Obes..

